# Integrative omics analysis reveals distinct adaptations of bongkrekic acid producing *Burkholderia gladioli pathovar cocovenenans* strains

**DOI:** 10.3389/fmicb.2025.1712709

**Published:** 2026-02-05

**Authors:** Jiale He, Lingguo Zhao, Yunke Sun, Qingliang Chen, Jiashu Li, Jun Chen, Yingdan Zhang, Liang Yang, Yang Liu, Lei Lei

**Affiliations:** 1 Medical Research Center, Southern University of Science and Technology Hospital, Shenzhen, China; 2 Joint Laboratory of Guangdong-Hong Kong Universities for Vascular Homeostasis and Diseases, Department of Pharmacology, School of Medicine, Southern University of Science and Technology, Shenzhen, China; 3 Bao'an District Center for Disease Control and Prevention of Shenzhen City, Shenzhen, China; 4 Shenzhen Third People's Hospital, The Second Affiliated Hospital of Southern University of Science and Technology, Shenzhen, China; 5 National Clinical Research Center for Infectious Disease, Shenzhen Third People's Hospital, Shenzhen, China

**Keywords:** bongkrekic acid, comparative genomic analysis, environmental adaptability, fluorescent probe detection method, omics analysis

## Abstract

**Introduction:**

*Burkholderia gladioli pathovar cocovenenans* is an emerging pathogen, with its key toxin, bongkrekic acid (BA), encoded by the *bon* gene cluster. BA has been linked to several fatal food poisonings. Although its toxicity is well-known, its ecological and evolutionary role in bacterial adaptation and pathogenicity remains unclear. This study aims to clarify the phylogenetic differentiation, genomic features, and ecological strategies of BA-producing strains, and develop rapid detection methods to reduce food safety risks.

**Methods:**

We performed a comprehensive genomic analysis of 305 *Burkholderia* strains, including 34 isolated strains and 271 publicly available genomes. Phylogenetic reconstruction traced the evolutionary trajectory, and comparative genomics identified genetic differences between BA-producing and non-producing strains. Transcriptomic and metabolomic profiles were generated under controlled conditions to compare gene expression and metabolic features. Specific probes were also designed for rapid detection of BA and toxoflavin.

**Results:**

Phylogenetic analysis revealed a clear differentiation between strains with and without the *bon* gene cluster, indicating that BA synthesis plays a crucial role in the evolution of *Burkholderia*. The acquisition of the *bon* gene cluster is hypothesized to have occurred at a later stage in evolution. Although the distribution of virulence factors was generally similar between the two groups, the *bon* gene cluster was associated with specific virulence traits, such as pyruvate metabolism and secretion systems. All strains harboured *β*-lactam antibiotic resistance genes within their genomes. Transcriptome and metabolome analyses revealed elevated expression of type III secretion system (T3SS)-associated genes in BA-producing strains, alongside increased organic acid and lipid metabolism. The newly developed probes enable specific, rapid detection of BA and toxoflavin, providing an effective tool for strain screening.

**Conclusion:**

Genomic, transcriptomic, and metabolomic analyses show that the *bon* gene cluster is highly conserved in *Burkholderia gladioli* and predominantly clustered within specific branches of the phylogenetic tree. The presence of this gene cluster correlates with specific pathogenic traits and is associated with alterations in T3SS expression and metabolic characteristics, which are closely linked to bacterial adaptability across diverse ecological environments. The developed toxin-specific probes provide a reliable tool for rapid detection, supporting future monitoring and control efforts.

## 1 Introduction

*Burkholderia gladioli pathovar cocovenenans (B. gladioli)* is both a plant pathogen and an opportunistic human pathogen. Initially recognized in the 1960s for infecting onions, irises, and ferns (Chase et al., 1984; Durgapal and Trivedi, 1977; Roberts, 1973), it was later linked to human pneumonia (Ross et al., 1995), and severe infections in immunocompromised patients (Hoare and Cant, 1996; Khan et al., 1996; Ross et al., 1995). Its public health significance emerged after a fatal food poisoning case in India (1951), leading to the identification of its lethal toxin, bongkrekic acid (BA) by Venn in 1966 (Buckle and Kartadarma, 1990). Recent epidemiological data indicate a marked increase in BA-related outbreaks, with mortality rates ranging between 40 to 100% (Han et al., 2023; Jiao et al., 2003; Zhang et al., 2023). To date, thousands of BA poisoning cases have been documented in regions across Africa, China, Southeast Asia, and North America, raising significant concerns regarding food safety and public health (Gudo et al., 2018; Su, 2024).

BA is a white solid with a molecular weight of 486 kDa. It remains structurally stable under high temperatures, making it difficult to eliminate through ordinary cooking methods (Anwar et al., 2017). BA is a potent respiratory toxin that impairs mitochondrial adenine nucleotide translocase (ANT), disrupting ATP/ADP exchange and leading to mitochondrial dysfunction (Haworth and Hunter, 2000; Lauquin and Vignais, 1976). It is recognized as one of the most potent respiratory toxins, with an LD50 value ranging from 1 to 3.16 mg/kg in humans, causing a high mortality rate upon ingestion of contaminated food (Henderson and Lardy, 1970; Ruprecht et al., 2019). Besides, BA has been widely employed as an apoptosis inhibitor in experimental studies (Teshima et al., 2003).

The production of BA in *B. gladioli* strains is known to be influenced by a range of environmental conditions, including pH, temperature, NaCl concentration, and fatty acid composition (Anwar et al., 2017; Moebius et al., 2012). Interestingly, sub-lethal concentrations of trimethoprim, a folate antagonist, have been found to inhibit BA production, while high glucose concentrations promote BA synthesis (Huang et al., 2025; Jones et al., 2021). These findings suggest that BA production may be regulated by a complex network of environmental signals and genetic factors.

The biosynthesis of BA is governed by the *bon* gene cluster, a contiguous 13-open reading frame (ORF) region, naming *bonA-M* (Anwar et al., 2017; Han et al., 2023; Moebius et al., 2012). *bonA-bonD* encode type I polyketide synthase (PKS) modules, *bonE-bonM* encode accessory components and enzymes mediating modification reactions, while the putative regulatory genes (*bonR1-R2*) are located upstream of *bonA-M* gene cluster (Anwar et al., 2017; Moebius et al., 2012). In some strains producing BA, such as *B. gladioli* Co14, the *bonC* ORF may be absent (Anwar et al., 2017). The *bon* gene cluster’s origin was studied in some *B. gladioli* strains, where IS transposases flanking *bonM* suggested horizontal gene transfer (HGT) acquisition (Peng et al., 2021). Another study suggested that pathogenic strains may show strictly conserved BA-related protein-coding sequences, distinct from non-BA-producing strains (Gong et al., 2023). However, large-scale comparative genomic and evolutionary analyses remain scarce.

Besides BA, *B. gladioli* also produce another potent toxin, toxoflavin, which induce plant lethality and severe mammalian toxicity (e.g., diarrhea, hemorrhaging, organ failure) (Anwar et al., 2017; Choi et al., 2023; van Damme et al., 1960). Its biosynthesis involves two gene clusters (synthesis and transport), with the latter regulated by the quorum sensing (QS) system *tofR-tofI* (Chen et al., 2012). Laboratory toxin detection primarily employs high-performance liquid chromatography (HPLC) and ultra-HPLC-mass spectrometry (uHPLC–MS). But simultaneous bongkrekic acid/toxoflavin quantification is hindered by their opposing solubility (lipophilic vs. water-soluble) (Gao et al., 2025). PCR targeting biosynthetic genes offers a promising alternative for dual-toxin strain screening. Comprehensive comparative genomics analysis is needed to optimize these assays, thereby providing accurate diagnostic and food safety risk assessment (Jiao et al., 2024; Moebius et al., 2012; Peng et al., 2021).

Despite the recognized lethality of BA in *B. gladioli* infections, the evolutionary drivers and ecological advantages conferred by the *bon* gene cluster remain poorly understood. In this study, we integrated comparative multi-omics analyses between *B. gladioli* strains with and without *bon* gene culsters (305 strains in total, including 34 novel isolates from food accidents). Comparative genomic analysis was performed to identify key differences in environmental adaptability. Phylogeny analysis was used to identified distinct evolutionary trajectories. Pathogenic and antibiotic resistance genes analysis was conducted to explore the genetic basis of environmental survival. Notably, by integrating transcriptomic and metabolomic data, key differences in gene expression and metabolic phenotypes between the two groups in response to environmental pressures was investigated. Besides, leveraging conserved genetic signatures, we developed rapid molecular probes for dual detection of BA and toxoflavin. This study not only deciphers the pathogen’s evolutionary strategies but also bridges fundamental research with practical interventions for outbreak prevention.

## 2 Materials and methods

### 2.1 Whole genome sequencing (WGS) analysis

From 2019 to 2023, we isolated 34 strains of *Burkholderia gladioli* from reported toxic rice noodle foods in Shenzhen, China. Culturing and isolation were performed in the reference laboratory of the Bao’an Center for Disease Prevention and Control in Shenzhen (details are shown in the Supplementary Table S1). The rice-derived products were streaked onto Potato Dextrose Agar (PDA) medium plates and incubated at 25 °C for 2–3 days. Round colonies, approximately 1–2 mm in diameter, exhibiting yellow pigment production, were then transferred to 20 mL of Potato Dextrose Broth (PDB) Medium and cultured at 25 °C for 24 h before genomic DNA extraction. For short-read sequencing, we extracted the genomic DNA of the 34 strains using the QIAamp DNA Mini Kit (QIAGEN, Hilden, Germany) and quantified with a Qubit fluorometer (ThermoFisher Scientific, Waltham, MA, United States). Short-read sequencing was performed on the Illumina Hiseq2500 platform, with a read length of 150 bp and a paired-end sequencing approach, achieving an average sequencing depth of 90x. Sequence filtering and trimming were carried out using the default parameters of FastQC v0.11.5 and Trimmomatic v0.32 (Bolger et al., 2014). Long-read sequencing was performed using the PacBio Sequel II platform (Pacific Biosciences, United States) or the MinION platform (Oxford Nanopore, UK). We used the cleandata from PacBio sequencing, converted the BAM files to FASTQ format using bedtools (Quinlan and Hall, 2010), and then performed hybrid assembly of long reads from PacBio and short reads from Illumina using Unicycler (Wick et al., 2017). This approach resulted in the generation of a complete genome assembly. For sequencing on the Nanopore platform, genomic DNA libraries were prepared with the sequencing kit SQK-LSK109 (Oxford Nanopore) and sequenced using a FLO-MIN106 flow cell. Genomic DNA libraries for sequencing on the Nanopore platform were prepared using the SQK-LSK109 sequencing kit (Oxford Nanopore) and sequenced with a FLO-MIN106 flow cell. The post-sequencing data were filtered using the default parameters of NanoFilt v2.8.0.^1^ A hybrid assembly of long MinION reads and short Illumina reads was performed using Unicycler v0.5.0 for both assembly and polishing of the hybrid sequences (Wick et al., 2017). The quality of the assemblies was evaluated using QUAST v2.3 (Gurevich et al., 2013).

Additionally, genome sequences for 271 *B. gladioli* strains available from NCBI as of September 2023 were downloaded for comparative genomics analysis. Annotation was performed using Prokka v1.14.6 (Seemann, 2014), which utilizes external prediction tools to identify genomic features such as CDS, tRNA, rRNA, CRISPR, and others mapped on overlapping groups and chromosomes. The command—genus Burkholderia was employed, resulting in the annotation of approximately 7,000 CDS per genome.

### 2.2 Phylogenetic analysis

The strain information used in this study is recorded in Supplementary Table S1. Phylogenetic analysis was conducted based on single nucleotide polymorphism (SNP) maps, employing the Parsnp v1.7.4 software (Treangen et al., 2014) from the Harvest bioinformatics toolkit—a utility enabling rapid multiple sequence alignment of core genomes. This tool facilitated SNP detection across 305 *B. gladioli* strain whole-genome FASTA files. The Parsnp command line was executed with the -c and -vcf parameters, generating the final SNP output file. The results were subsequently input into Ramxl (v8.2.13) to construct a phylogenetic tree, undergoing 1,000 ultrafast bootstrap iterations. Visualisation and optimisation of the evolutionary tree were performed using iTOL v6 (Letunic and Bork, 2024), integrating appropriate metadata to enhance interpretability and visual presentation of the phylogenetic relationships.

Phylogenetic relationships within the core genome were inferred using OrthoFinder (v3.1.0), excluding the *bon* gene cluster and other annotated mobile genetic elements. Subsequently, each core gene was aligned using MAFFT (v7.526) under default parameters. The resulting alignments were concatenated and input into IQ-TREE (v3.01) to construct a maximum likelihood phylogenetic tree.

### 2.3 Analysis of the *bon* gene cluster

The reference sequence for the *bon* gene cluster, identified in *Burkholderia cocovenenans* DMSZ11318, was obtained from NCBI (Accession number: JX173632). Alignment of the whole genome FASTA files from 305 *B. gladioli* strains with this reference sequence was performed using the nucmer module of the MUMmer software (v3.23) (Riva and Mauri, 2021), with default parameters set. The dnadiff module was utilized to process the raw alignment files, and the show-coords module was used to query the alignment result files. The results showed that the *bon* gene cluster was fully or partially detected in 22 strains isolated in this study and 36 strains retrieved from NCBI. Among the latter, the complete *bon* gene cluster was located on a single contig in 21 strains.

Furthermore, based on the blastp alignment results against the reference sequence, *bon* proteins with identity > 90% were screened from the protein sequences of 304 strains annotated using Prokka (v1.14.6). The conservation and integrity of the Pfam domains of these *bon* proteins were verified via the hmmscan function of HMMER (v3.4), the parameter E-value was set to 1e-5 to filter and obtain the final results.

Subsequently, the ffn sequences of these 43 *B. gladioli* strains were compared against the reference sequence (JX173632) using blastn (Seemann, 2014). The blastn results were processed and overlapping sequences were extracted using custom scripts. Additionally, pyGenomeviz^2^ was employed to generate visualizations depicting the homology of the *bon* gene cluster.

### 2.4 Detection of BA produced by *Burkholderia gladioli* using UPLC-MS/MS

Approximately 5.00 g of rice-derived products was mixed with an ammonia-methanol solution for ultrasonic extraction. The samples were then centrifuged at 4 °C, and the supernatant was filtered through a 0.2 μm membrane filter for analysis. Chromatographic separation was performed using a Waters ACQUITY UPLC CSH C18 column, with a gradient elution method employing water with 0.2% acetic acid (mobile phase A) and acetonitrile with 0.2% acetic acid (mobile phase B). Mass spectrometric analysis was conducted in negative ion mode with electrospray ionization (ESI) and multiple reaction monitoring (MRM). BA standard solutions, purchased from Shanghai Anpu Experimental Technology Co, Ltd. and Beijing Tanmo Quality Inspection Technology Co, Ltd., were diluted to prepare working solutions of various concentrations to construct standard curves for BA quantification. The calibration curves were used to correlate peak areas to concentrations, and quantification was achieved using a weighted least-squares linear regression analysis. The limit of detection (LOD) and limit of quantification (LOQ) were determined by the signal-to-noise method, using a ratio of 3:1 for LOD and 10:1 for LOQ (Zhao et al., 2025).

### 2.5 Prediction and analysis of virulence factors and resistance genes

A local pathogen database was constructed based on the Virulence Factor Database (VFDB, setA) available at VFDB: Virulence Factor Database (Chen et al., 2005). This setA database constitutes the core collection of the VFDB and was employed to analyze potential virulence-related genes across 305 *B. gladioli* strains. Predictions were conducted using the DIAMOND v2.1.8 software with the BLASTP function, applying parameters of –id 50 and -e 1e-5 for identification and significance thresholds, respectively. Heatmaps illustrating the distribution and prevalence of these genes were generated using the pheatmap package (v1.0.12) and ggplot2 package (v3.4.4) in R version 4.3.2.

We used a custom R script to analyze the potential correlation between *bon* gene cluster and virulence factors or antibiotic-resistance genes. Spearman correlation analysis was performed to generate a correlation matrix, and the results were visualized using the pheatmap package (v1.0.12). Additionally, predictions for antibiotic resistance genes and efflux pump family genes were performed on FASTA-formatted files using the default parameters of AMRFinderPlus v3.11.26,^3^ which utilizes refined Hidden Markov Models for screening.

### 2.6 Phylogenetic and structure analysis of ANT homologs

Human adenine nucleotide translocase (ANT/SLC25A4, accession number: NP_001142.2) was retrieved from the NCBI GenBank database. The FAA sequence of this protein was used to perform a BLASTP search against the nr/nt database with the online BLAST tool, selecting bacteria (taxid: 2) and retrieving the alignment results along with homologous protein sequences. Multiple sequence alignment of the homologous proteins was conducted using MAFFT (v7.525) (Katoh and Standley, 2013), followed by phylogenetic tree construction using the maximum likelihood method in IQ-TREE (v2.3.6) (Nguyen et al., 2015). The resulting tree was visualized and annotated using iTOL (v6) (Letunic and Bork, 2024). The 3D structure alignments of the proteins were performed using PyMOL (v3.1.3).

### 2.7 Transcriptomics analysis

We selected two BA-producing toxigenic strains, BAJK01 and BAJK03, and two non-BA-producing strains, BAJK04 and BAJK22, for transcriptome sequencing. The four selected strains were cultured overnight and then transferred to fresh PDB medium for cultivation at 37 °C until the OD600 reached 0.6–0.8. Total RNA was immediately extracted using TRIzol reagent (CWBIO, China) according to the manufacturer’s instructions. The extracted RNA was quantified using Qubit 2.0 (Thermo Fisher Scientific, MA, United States) and Nanodrop One (Thermo Fisher Scientific, MA, United States). RNA libraries were constructed following the standard protocol of the NEBNext^®^ Ultra^™^ Directional RNA Library Prep Kit for Illumina^®^ (New England Biolabs, MA, United States). Ribosomal RNA was removed using the Ribo-zero rRNA Removal Kit. cDNA synthesis was performed with the NEBNext First Strand Synthesis Reaction Buffer. Paired-end RNA sequencing (150 bp read length) was performed on the Illumina NovaSeq PE150 platform.

The RNA-seq reads were preprocessed and mapped to Annotated transcripts of the reference genome using kallisto v0.51.0 (Bray et al., 2016). Differentially expressed genes (DEGs) were identified using DESeq2 v1.42.1 (Love et al., 2014), based on the negative binomial distribution. The Wald test was used to calculate *p-*values, which were then adjusted for multiple testing using the Benjamini-Hochberg procedure to control the false discovery rate (FDR). Genes with an adjusted *p*-value < 0.05 and |log2FoldChange| > 1 were identified as differentially expressed genes (DEGs). We set the parameters as |log2FC| > 1 and adjusted-*p* value < 0.05 to filter the genes for the subsequent enrichment analysis. Gene Ontology (GO) enrichment analysis and Kyoto Encyclopedia of Genes and Genomes (KEGG) enrichment analysis were conducted using clusterProfiler v4.10.1, with the reference species set as “bgd” (Wu et al., 2021). Principal component analysis (PCA) plots, volcano plots, and enrichment analysis plots were generated using ggplot2 v3.5.1, while heatmaps were created using pheatmap v1.0.12.

### 2.8 Quantitative real-time PCR (qRT-PCR) validation

To validate the RNA-seq data, four bacterial strains (BAJK01, BAJK03, BAJK04, BAJK22) were cultured to an OD600 of 0.6–0.8. Total RNA was extracted and reverse-transcribed into cDNA using DP430 and KR136 (TIANGEN, China) according to the manufacturer’s instructions. qRT-PCR was performed on a Bio-Rad CFX96 Real-Time PCR System using Hieff UNICON^®^ Advanced qPCR SYBR Master Mix (Yeasen, China). *recA* was used as the internal reference gene for normalization. Relative gene expression levels were calculated using the 2^-ΔΔCt^ method. Statistical analysis was performed using one-way analysis of variance (ANOVA) followed by Tukey’s *post hoc* test for multiple comparisons. All experiments were performed in triplicate.

### 2.9 Mass spectrometry-based comparative metabolomics profiling

The BA-producing strains BAJK01 (T1) and BAJK03 (T2), as well as the non-BA-producing strains BAJK04 (NT1) and BAJK22 (NT2), were inoculated into 10 mL of PDB medium and incubated at 26 °C for 10 days. All experiments were performed in six biological replicates per strain. After inoculation, the cultures were centrifuged at 14,000 rpm for 10 min at 4 °C. 200 μL of the supernatant was mixed with 600 μL of methanol, and then centrifuged again at 14,000 rpm for 10 min at 4 °C. 200 μL of the supernatant was transferred to a sample vial for further UHPLC-Q-Orbitrap HRMS analysis. To ensure data quality in the metabolomic analysis, quality control (QC) samples were prepared by pooling 50 μL of medium from each sample. The QC samples were treated according to the procedure described above and injected at both the beginning and end of the analysis sequence.

Untargeted metabolite analysis was performed using a Thermo Scientific Vanquish Flex UHPLC system coupled to an Orbitrap Exploris 240 high-resolution mass spectrometer (Thermo Scientific, Bremen, Germany), equipped with a heated electrospray ionization (ESI) source operating in both positive and negative ion modes. Chromatographic separation was achieved on a Waters ACQUITY UPLC HSST3 column (2.1 × 100 mm, 1.8 μm) with the column temperature maintained at 40 °C and a mobile-phase flow rate of 0.30 mL/min. The mobile phases consisted of ultra-pure water with 0.01% (v/v) formic acid (A) and acetonitrile (B), gradient elution: 0–2 min, 5% B; 2–12 min, 5–95% B; 12–17 min, 95% B; 17–18 min, 95–5% B; 18–20 min, 5% B, with an injection volume of 10 μL. The optimized MS parameters were as follows: ion spray voltage, 3.5 kV (positive) and 2.5 kV (negative); sheath gas flow rate, 35 arb; auxiliary gas flow rate, 7 arb; sweep gas flow rate, 1 arb; vaporizer temperature, 325 °C; HCD collision energy (%), 15, 30, and 45. Data were acquired using the full MS/dd-MS2 approach in both positive and negative ion modes. Full scan spectra and MS/MS data were collected with resolutions of 60,000 and 30,000 FWHM, respectively.

Compound Discoverer 3.3 software (Thermo Fisher Scientific, United States), in conjunction with ChemSpider, MassLists, mzCloud, mzVault, and local databases to identify compounds. The resulting data matrix was imported into SIMICA software (version 14.1, Umetrics, Sweden), including unsupervised principal component analysis (PCA) and orthogonal partial least squares discriminant analysis (OPLS-DA). OPLS-DA was used to identify discriminative features between the BA-producing and non-BA-producing groups, and a permutation test was conducted 200 times to assess the risk of overfitting for the OPLS-DA model. For univariate analysis, statistical significance between groups was determined using an unpaired two-tailed Student’s t-test, the resulting *p*-values were adjusted for multiple comparisons using the Benjamini-Hochberg false discovery rate (FDR) method. Differential features were selected based on a variable importance in projection (VIP) value > 1.0 and an adjusted *p*-value < 0.05. Hierarchical clustering heatmaps were generated using Ward’s method and Euclidean distance. Additionally, Volcano plots were used to select differential features based on adjusted *p*-values and fold change (FC) values (Li et al., 2024).

### 2.10 Multi-omics guided design of TaqMan probes

Since *bonA* is the key gene involved in the production of BA, and sequence alignment reveals that *tofI* is more conserved than *tofR*, we selected these two genes as targets for detection. The *bonA* gene from *Burkholderia gladioli pathovar cocovenenans* DMSZ1131 and the *tofI* gene from *Burkholderia gladioli* BSR3 were used as reference sequences (Lee et al., 2016; Moebius et al., 2012), and a BLASTP search was performed across the genomes of 305 *B. gladioli* strains. The extracted sequences were then subjected to multiple sequence alignment, which identified conserved regions within the *bonA* and *tofI* genes. Based on these conserved regions, qPCR primers were designed for the experiment. DNA was extracted from 12 *B. gladioli* strains and diluted 10-fold to serve as templates for quantitative polymerase chain reaction (qPCR) assays targeting the expression of BA and Toxoflavin (see Table 1).

**Table 1:** Primers and probe sets designed to target *bonA* and *tofI.*

Primer name	Primer sequence (5′–3′)	Target
*bonA*-F	TGCGTCATCGGCAGATTC	Bongkrekic acid (BA)
*bonA*-R	CCTTCAGCTCGGTATTGACAT	
*bonA*-P	5`6-FAM-CTGCATTTCGAGCAGGGCAACC- 3’MGB	
*tofI*-F	GCGACTATTGCCGACCAC	Toxoflavin
*tofI*-R	ATTCCCACACTTCGGGAGA	
*tofI*-P	5`6-FAM-TACCTGCTGCACGAGGTGTTCC- 3’MGB	

The total reaction volume for qPCR was 20 μL, consisting of 10 μL of 2 × Premix Ex Taq (TAKARA, Dalian, China), 0.5 μL of each primer (10 μmol·L − 1) for the target genes, 1 μL of the probe, 2 μL of DNA template, and 8.5 μL of ddH2O, with ultrapure water used as a negative control. qPCR experiment was performed on a LightCycler^®^ 96 Real-Time PCR System (Roche, Branchburg, NJ, United States) with the following conditions: an initial denaturation at 95 °C for 30 s, followed by 40 cycles of denaturation at 95 °C for 5 s and extension at 63 °C for 20 s. To ensure the consistency and reliability of the results, all qPCR experiments were performed in triplicate.

In the sensitivity assay, we extracted genomic DNA from BAJK01 (26.3 ng/μL) and diluted it tenfold, a hundredfold, and a thousandfold, respectively. In the specificity assay, we employed three verified toxin-producing strains of *B. gladioli*, three non-BA-producing strains of *B. gladioli,* and one strain of *Pseudomonas aeruginosa*. DNA was extracted from each and subjected to probe-based qPCR.

## 3 Results

### 3.1 Genomic assembly and phylogenetic analysis of *Burkholderia gladioli*

In this study, 34 local *B. gladioli* strains were sequenced and assembled by using a combination sequencing strategy of Illumina and Oxford Nanopore platforms. The assemblies were evaluated using QUAST v5.1.0, revealing an average GC content of 68% and an average N50 length of 3630992 bp, with genome fraction consistently above 88%. The genome sizes of the *B. gladioli* strains ranged from 8.27 Mbp to 8.99 Mbp, featuring 1 to 2 chromosomes and 0 to 4 plasmids, indicating high adaptability to various environmental and survival conditions. Genome annotation revealed an average of 7,216 genes per strain, with the highest gene count reaching up to 7,643.

**Figure 1:**
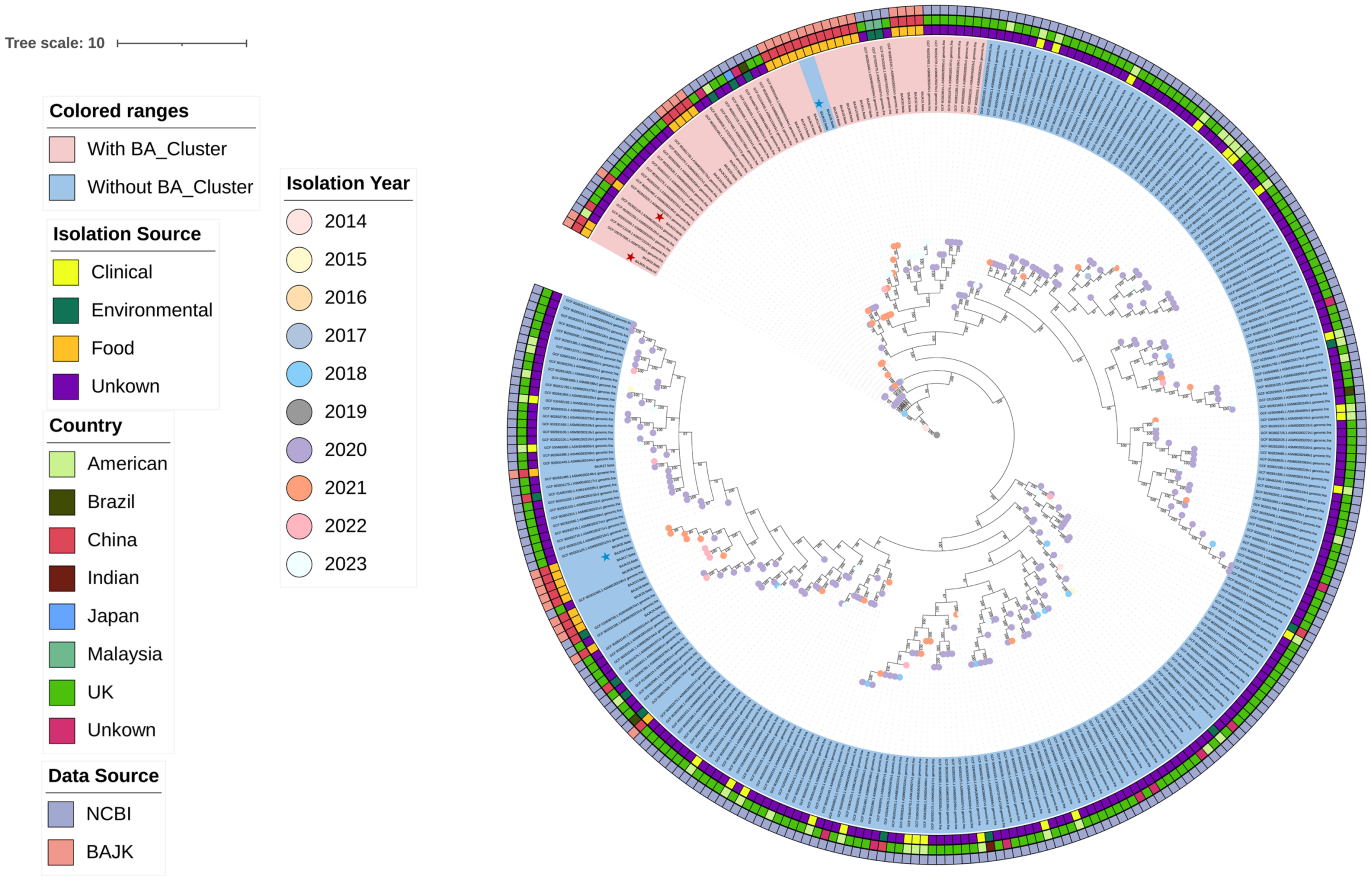
Phylogenetic tree of *B. gladioli.* This phylogenetic tree represents a comprehensive analysis of 305 *B. gladioli* strains, including 34 strains isolated in this study and 271 strains from NCBI GenBank. The information in the tree, from innermost to outermost, displays the isolation year, toxigenic status, isolation source, country of origin, and data source of these strains. Four *B. gladioli* strains, marked with red (BA-producing) and blue (non-BA-producing) stars, were selected for subsequent multi-omics analysis.

The phylogenetic tree of 305 *Burkholderia gladioli* strains in Figure 1 reveals a clear evolutionary divergence between strains harbouring the *bon* gene cluster (marked in pink) and those lacking it (marked in blue). Strains containing the *bon* gene cluster (pink-colored) are primarily concentrated in multiple clades within the phylogenetic tree, rather than forming a single continuous phylogenetic lineage. Notably, in some clades dominated by *bon*-containing strains, a small number of *bon*-negative strains are still observed to be nested within them, indicating that the presence of the *bon* gene cluster is not entirely consistent among strains with highly close phylogenetic relationships. Furthermore, from the overall structure of the tree, *bon*-containing strains are mostly distributed in the terminal clades of the phylogenetic tree. Different colored circles on the tree branches indicate the isolation year of each strain. The innermost circle provides information about the isolation source, indicating whether the strain was isolated from food, the environment, or clinical settings. Unfortunately, due to incomplete information provided by GenBank, many sources remain unidentified. However, it is still apparent that no toxigenic strains were isolated from clinical sources. The second inner circle is color-coded based on the country of sample origin, showing that most of the existing *B. gladioli* genome data originates from the UK. The outermost circle displays the data source for these 305 strains, either from NCBI or this study (BAJK). In addition, the figure highlights four strains with star-shaped markers, which represent the selected BA-producing strains (pink) and non-BA-producing strains (blue) used for subsequent transcriptomic and metabolomic analyses.

To evaluate whether the *bon* gene cluster and other mobile genetic elements (MGEs) affect phylogenetic inference, a core genome phylogenetic tree was reconstructed based on core genome sequences after excluding the *bon* gene cluster and associated mobile genetic elements (Supplementary Figure S1). The topology of previously constructed phylogenetic tree was nearly identical to that of the whole-genome phylogenetic tree, with no changes observed in the relative positions of *bon*-containing and *bon*-negative strains.

### 3.2 Comparative genomics analysis of the bongkrekic acid gene cluster

In constructing the phylogenetic tree for *B. gladioli*, a distinct divergence was observed between strains that contain the *bon* synthesis gene cluster and those that do not. This observation prompts further investigation into the stability and potential evolution pattern of *bon* gene clusters.

In the preliminary steps, we performed BLASTP alignment using the reference sequence of the canonical *bon* gene cluster (Accession: JX173632). The results confirmed that among the 305 *B. gladioli* strains, a total of 56 strains harbored the *bon* gene cluster, of which 43 strains contained the complete gene cluster located on a single contig.

To exclude interference from non-sense mutations, frameshift mutations, and other variations affecting the *bon* gene cluster’s function, we examined the Pfam domain conservation and integrity of the proteins corresponding to the 12 open reading frames (ORFs) in these strains, comparing them against reference protein sequences. HMMER analysis results confirmed that nearly all open reading frames in these *bon* gene cluster-bearing strains retained their predicted Pfam domains (see Table 2; Supplementary Table S2). Notably, no domains were predicted for the *bonE* protein. Furthermore, while homologous proteins such as *bonC* and *bonD* were detectable in some strains, these proteins lacked complete domains.

**Table 2:** Pfam domain conservation summary of the 12 ORFs in the *bon* gene cluster.

ORF	Expected Pfam domain	% strains with intact domain
bonA	PF00109, PF02801, PF08659, PF00550, PF14765, PF16197, PF00108, PF00106, PF13561, PF08242, PF13489, PF13649, PF08241, PF13847	Total = 58, intact = 54 (93.1%)
bonB	PF00109, PF14765, PF08659, PF02801, PF16197, PF00550, PF00106, PF13561, PF00108	Total = 58, intact = 58 (100.0%)
bonC	PF00109, PF14765, PF02801, PF08659, PF16197, PF00550, PF00106, PF00108	Total = 60, intact = 58 (96.7%)
bonD	PF00109, PF02801, PF00550, PF08659, PF14765, PF16197, PF00107, PF13602, PF00108, PF08242, PF13649, PF13489, PF08241, PF00106, PF08240, PF13847, PF13561	Total = 60, intact = 52 (86.7%)
bonF	PF00109, PF02801	Total = 58, intact = 58 (100.0%)
bonG	PF08540, PF01154, PF00195	Total = 58, intact = 58 (100.0%)
bonI	PF00378, PF16113	Total = 64, intact = 58 (90.6%)
bonJ	PF00698	Total = 58, intact = 58 (100.0%)
bonK	PF00698	Total = 58, intact = 58 (100.0%)
bonL	PF00067	Total = 58, intact = 58 (100.0%)
bonM	PF13649, PF08241, PF02353, PF01209, PF13489, PF13847	Total = 58, intact = 58 (100.0%)

**Figure 2:**
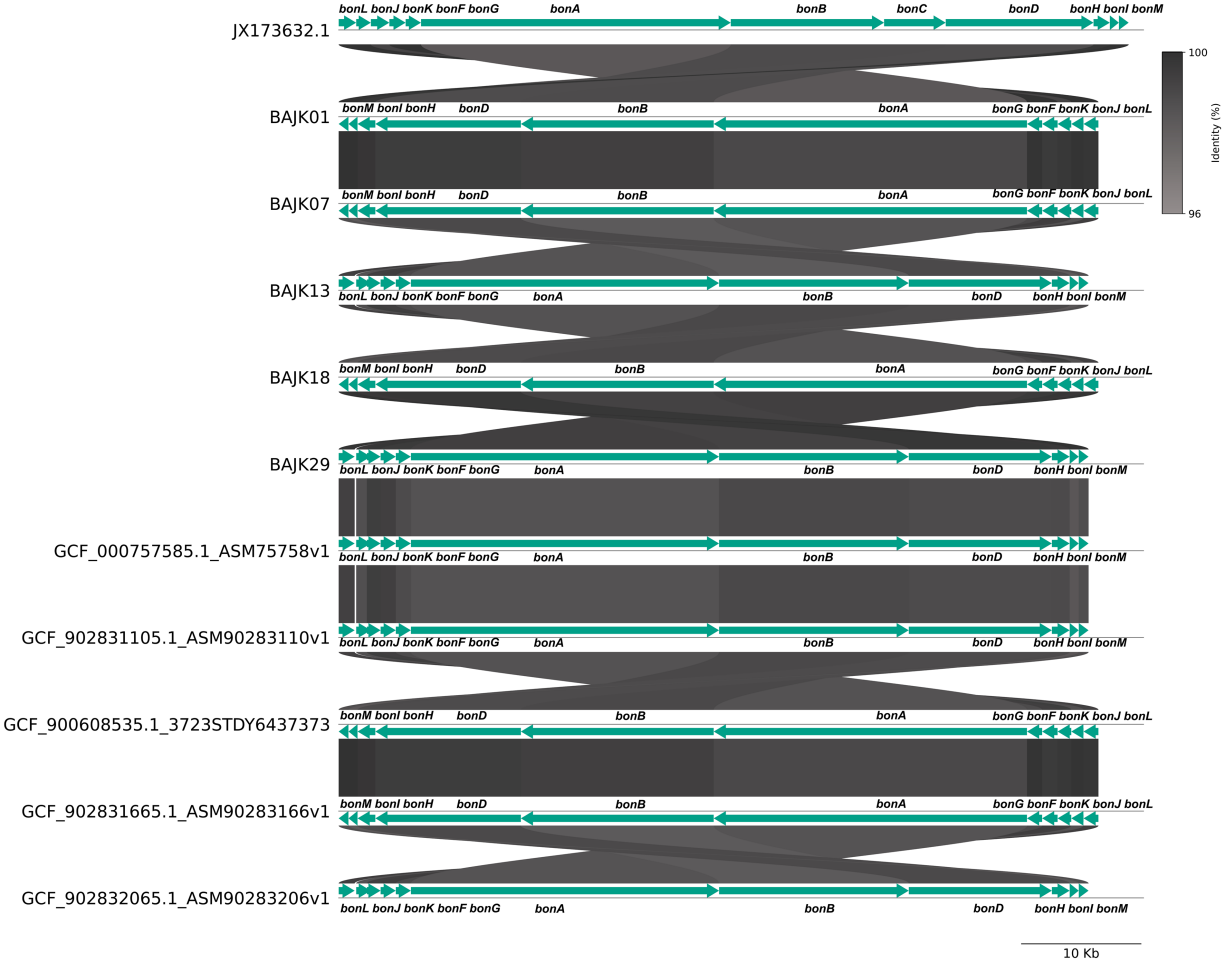
Collinearity analysis of the *bon g*ene cluster of different *B. gladioli* strains. This figure illustrates the collinearity analysis of the *bon* gene cluster. Gene names are annotated above the arrows, which indicate the direction of the genes. The shaded areas represent the homology between the genes, highlighting regions of genetic similarity across different strains.

Subsequently, using pyGenomeviz (Python v0.4.4), collinearity comparisons were performed between the standard *bon* gene cluster (Accession number: JX173632) and the 43 extracted *bon* gene cluster sequences (Supplementary Figure S2). For visualization, five of these *bon* gene cluster sequences were randomly selected based on their source—either from NCBI database or from the current sequencing data—and subjected to a collinearity comparison (Figure 2). The results revealed a high degree of conservation (identity ≥96%) within the *bon* gene cluster.

### 3.3 Association between the *bon* gene cluster and virulence genes in *Burkholderia gladioli*

*Burkholderia gladioli* strains capable of producing BA can cause serious infections in both humans and rice crops. We hypothesized that, in addition to acquiring the *bon* gene cluster, these strains might have developed other virulence mechanisms during evolution. To investigate whether the presence of the *bon* gene cluster influences the distribution of virulence factors in *B. gladioli*, we analyzed 305 strains using the DIAMOND software with the VFDB setA database, applying a sensitivity threshold of id ≥ 50%. A total of 309 virulence-related genes were identified, grouped into 12 categories (Supplementary Table S3). Among these, genes associated with motility and immune modulation were the most common, with 64 and 61 genes identified, respectively. These genes play roles in functions such as chemotaxis, motility, Type III secretion systems (T3SS), hemolysins, lipopolysaccharide synthesis, and immune evasion. Additionally, urease-related genes *ureA* and *ureB* were detected, and these genes are typically associated with microbes’ ability to regulate the pH of their surrounding environment.

**Figure 3:**
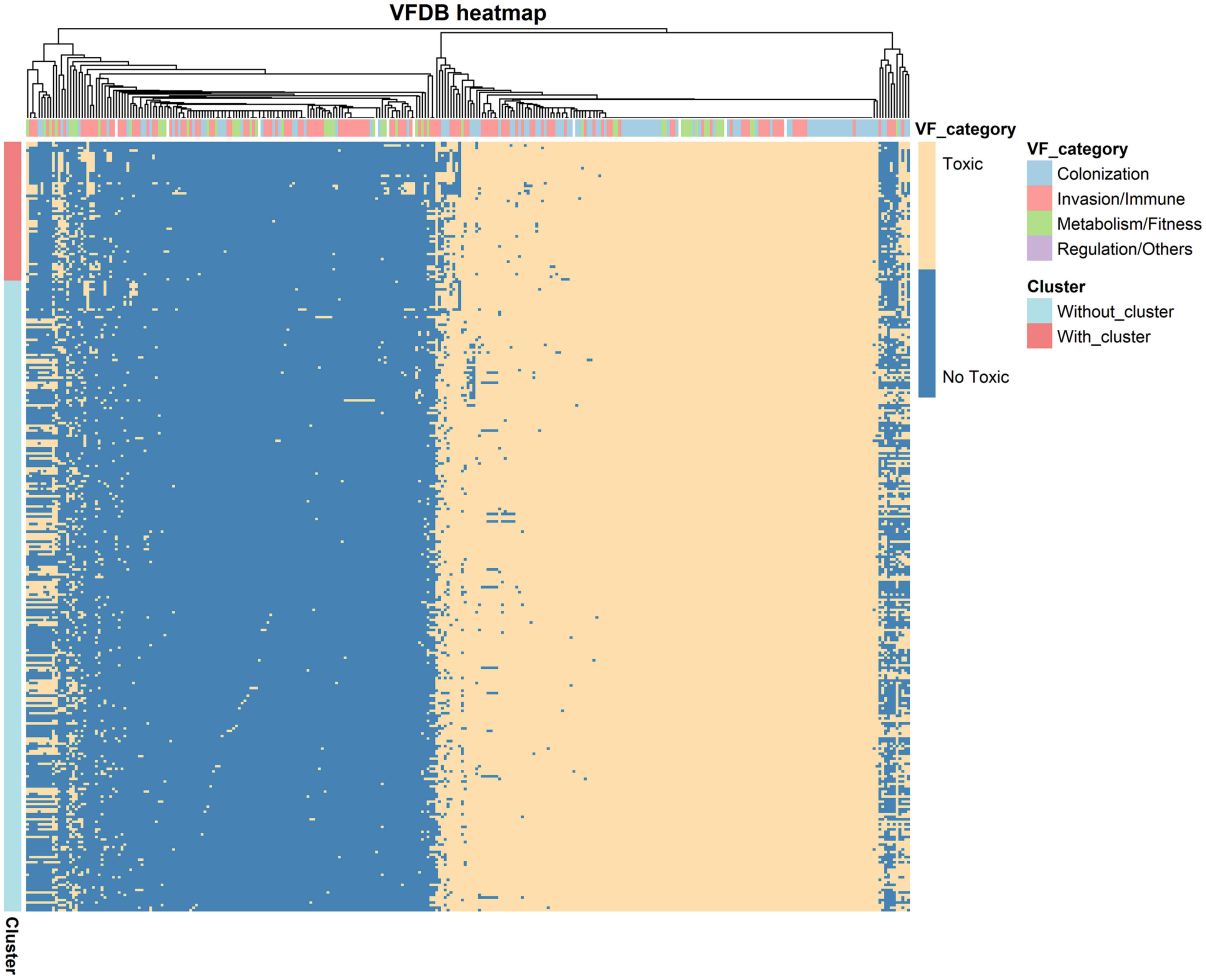
Heatmap of Virulence Factors in *B. gladioli* strains. The heatmap illustrates the distribution of virulence factors in *B. gladioli*, differentiated by the presence or absence of the *bon* gene cluster. Yellow indicates the presence of a gene, while blue denotes its absence. The vertical axis labels represent whether the *bon* gene cluster is present or not, and the horizontal axis labels categorize the virulence factors.

To visualize the distribution of virulence genes across the 305 strains, we constructed a heatmap (Figure 3), where columns represent virulence genes and rows represent strain names. The heatmap showed a clear distribution pattern of virulence factors, particularly the consistency of core virulence genes in the Motility and Immune Modulation categories, regardless of whether strains carried the *bon* gene cluster. This finding suggests that *B. gladioli* may have retained these genes throughout its evolution to adapt to various environments and hosts.

Further analysis using spearman correlation (threshold: ±0.3, *p* < 0.05) revealed that while the presence of the *bon* gene cluster did not significantly alter the overall distribution of virulence genes, some specific virulence factors showed notable correlations with its presence. Genes negatively correlated with the *bon* gene cluster included those involved in secretion systems (*iagB, vasH*), iron metabolism (*pvdH*), and alginate synthesis (*algA*). In contrast, genes positively correlated with the *bon* gene cluster included those linked to Type VI secretion systems (*tssC*) (Förster et al., 2014), nitrate reduction (*narH*), pyruvate metabolism (*ppsA*), and polyketide synthesis (*pks15*) (Supplementary Figure S3A).

### 3.4 Association between the *bon* gene cluster and antibiotic resistance genes in *Burkholderia gladioli*

**Figure 4:**
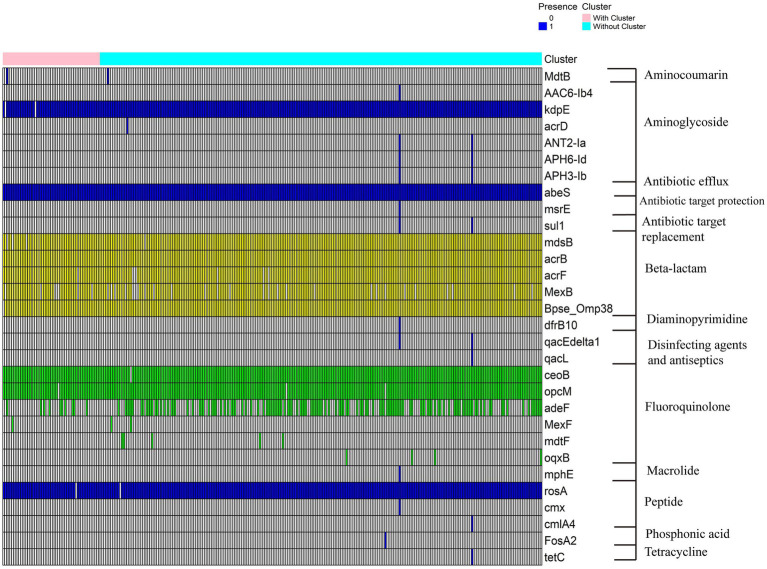
Heatmap of antibiotic resistance genes. The heatmap illustrates the presence and absence of antibiotic resistance genes across different *B. gladioli* strains. Blue cells indicate the presence of a gene in a given strain, while white cells indicate its absence. Genes classified as *β*-lactam resistance genes and fluoroquinolone resistance genes are highlighted in yellow and green, respectively. Strain names highlighted in orange represent isolates obtained in this study (BAJK), whereas those in black were retrieved from NCBI.

By comparing the genomes of 305 *B. gladioli* strains with the Comprehensive Antibiotic Resistance Database (CARD) (Supplementary Table S4), we identified their antibiotic resistome. A total of 30 antibiotic resistance genes (ARGs) were detected across these strains, with most genes being highly conserved in *B. gladioli* (Figure 4). For example, efflux pump-related genes such as *abeS* and *mdsB*, as well as well-known resistance genes like *msrE* and *dfrB10*, were found in many strains. Notably, our study suggests that *B. gladioli* may possess intrinsic *β*-lactams activity, as the *bpse_Omp38* gene was consistently present in all analyzed strains, regardless of their source. *bpse_Omp38* is a β-lactam resistance gene unique to the *Burkholderia* genus. Unlike traditional β-lactamases, it likely confers resistance by modulating porins, reducing β-lactam permeability and thereby limiting antibiotic entry. This indicates that *B. gladioli* may have evolved a natural resistance mechanism against β-lactams, which persists even in the absence of external antibiotic pressure.

In contrast, some ARGs, such as sul1, *AAC(6′)-Ib4*, *APH(6)-Id*, *msrE*, and *tetC*, were only found in certain strains. This suggests that their presence may be linked to environmental selection pressures. Strains isolated from environments with prolonged exposure to sulfonamides, aminoglycosides, macrolides, or tetracyclines may have retained these resistance genes through adaptive evolution. Efflux pump systems also play a crucial role in *B. gladioli* resistance. Multiple efflux-related genes were identified, including *mdsB* and *acrB*, which are associated with β-lactam resistance but also mediate resistance to various other antibiotics. This suggests that efflux pumps contribute significantly to the complex resistance profile of *B. gladioli*, enhancing its multidrug resistance (MDR) and overall fitness.

To further investigate the relationship between antibiotic resistance and *bon* gene cluster, we compared the predicted resistome of *B. gladioli* based on the presence or absence of *bon* gene cluster, a key genomic locus associated with BA biosynthesis. No significant differences in resistance profiles were observed between strains with or without *bon* gene cluster. This finding suggests that *bon* gene cluster is not directly associated with antibiotic adaptation. Furthermore, we conducted Spearman correlation analysis between the *bon* gene cluster and antibiotic resistance genes to identify potential associations (Supplementary Figure S3B). However, no significant correlations were observed, as all genes exhibited correlation coefficients with absolute values below the threshold of 0.4.

### 3.5 Potential targeting of ANT homologs in bacteria by bongkrekic acid

Previous studies have shown that the target of BA is the ATP/ADP translocase (ANT) in the mammalian mitochondrial respiratory chain. BA induces cell dysfunction by inhibiting conformational changes in ANT, ultimately leading to the death of infected individuals (Ruprecht et al., 2019). To investigate whether BA might act through a similar mechanism to kill bacteria, we selected the human SLC25A4 protein (accession number: NP_001142.2) as a target and conducted a BLASTP search for homologous proteins in bacteria. The results revealed that homolog protein of SLC25A4 is only found in certain bacterial species. Among them, The two proteins with the highest homology, 80.77 and 83.1, respectively, were derived from *Escherichia coli*. To further analyze these homologous proteins, we downloaded all successful aligned sequences and performed multiple sequence alignments using MAFFT software. Subsequently, we constructed a phylogenetic tree using IQ-TREE (Supplementary Figure S4A). A heatmap showing the homology of these 50 homologous proteins was included in the tree. The results showed that some proteins had homology as low as 30%, suggesting that ANT homologs are not universally present in bacteria. Nonetheless, only 9 sequences in the phylogenetic tree exhibited approximately 80% homology and displayed conserved regions, indicating that they may share key structural domains. Additionally, we used PyMOL to perform 3D structural alignment of the two *E. coli* proteins with SLC25A4. The RMSD values for the alignments were 0.389 (WP_266143627.1) and 0.410 (WP_266145757.1), indicating that the two *E. coli* proteins have a high degree of structural similarity with SLC25A4, with similar spatial folding and atomic positioning (Supplementary Figure S4B). These findings provide preliminary structural evidence for the potential of BA to act through a similar mechanism in bacteria.

### 3.6 Integrative transcriptomics analysis of BA-producing and non-producing strains

**Figure 5:**
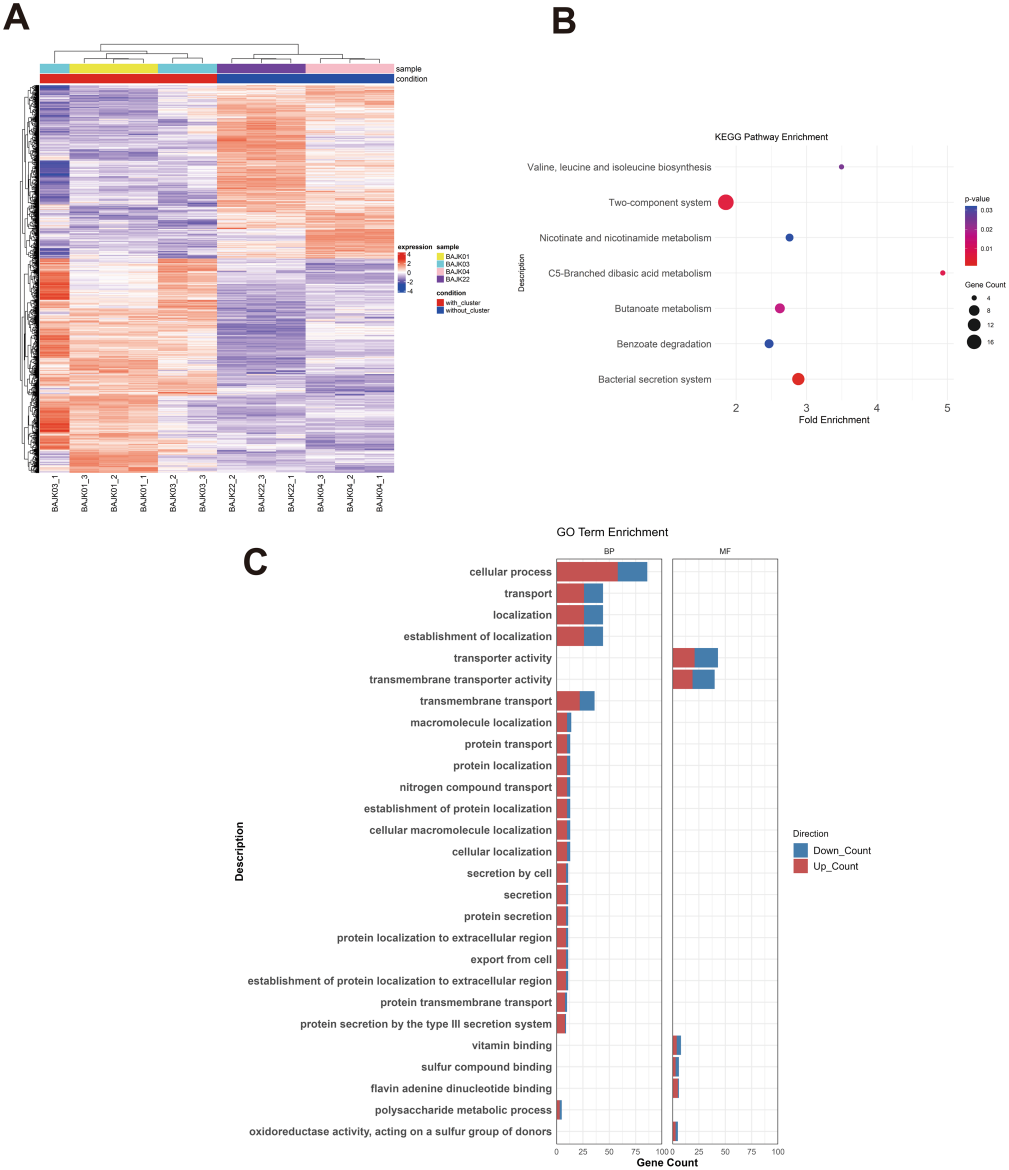
RNA-seq analysis of isolates containing and lacking the *bon* gene cluster. **(A)** A heatmap of DEGs is shown, with three biological replicates performed for each sample. **(B)** GO enrichment analysis of DEGs, *p*-value < 0.05. BP: Biological process; MF: molecular function. The size of the circles represents the number of genes enriched in the pathway. **(C)** KEGG enrichment analysis of DEGs, bar with red and blue indicating genes assigned to the functions were upregulated or downregulated.

To investigate whether transcriptional differences are associated with the presence of the *bon* gene cluster between BA-producing and non-BA-producing *Burkholderia gladioli* strains, we selected four *B. gladioli* strains for RNA sequencing (RNA-seq) analysis on the Illumina HiSeq platform. These four strains are marked with star symbols in Figure 1, with the red stars indicating the BA-producing strains (BAJK01, BAJK03) and the blue stars representing the non-BA-producing strains (BAJK04, BAJK22). The four strains are phylogenetically distinct, providing a comprehensive approach to examine the impact of the BA cluster on the environmental adaptability of *B. gladioli* strains. This comparative transcriptomic approach focused on identifying differentially expressed genes (DEGs) between strain groups to elucidate molecular mechanisms underlying their ecological adaptations. By applying a filtering threshold of absolute fold change ≥ 1 and *p*-value < 0.05, a total of 419 DEGs were identified in isolates containing the *bon* gene cluster compared to those without. Among these, 287 genes were upregulated, while the remaining genes were downregulated. A heatmap of the DEGs revealed a distinct transcriptional profile associated with the presence or absence of the *bon* gene cluster (Figure 5A). Additionally, the clear separation of isolates with and without the *bon* gene cluster along the PC1 axis, which accounts for 46% of the total variance, suggested significant physiological differences between these groups (Supplementary Figure S5).

Subsequently, researchers conducted GO functional enrichment analysis and KEGG pathway enrichment analysis on differentially expressed genes to identify enriched functions potentially associated with the *bon* gene cluster in the strains (Figures 5B,C). The GO enrichment analysis revealed that, compared to strains lacking the *bon* gene cluster, strains containing this cluster exhibited enrichment across 27 GO functional entries, comprising 21 biological process entries and 6 molecular function entries. Notably, the most highly enriched biological process was protein secretion associated with the type III secretion system (T3SS) (see Figure 5B), involving genes such as *sctS, sctR, ssaV, sctN, sctT*, the *BGLA_RS20400* gene encoding a T3SS chaperone protein (belonging to the *CesT* family), and *hrpB2*. Except for *BGLA_RS20400*, which exhibited a 3.07-fold downregulation, the remaining genes showed upregulation ranging from 2-fold to 3.71-fold. To validate the RNA-seq findings, we selected three genes associated with the type III secretion system based on enrichment results for qRT-PCR analysis. As shown in Supplementary Figure S6, BAJK01 and BAJK03, which harbour the *bon* gene cluster, exhibited significantly higher expression levels for all three genes compared to BAJK22 and BAJK04 strains lacking the *bon* gene cluster, consistent with the transcriptome sequencing results. Additionally, transmembrane transport was enriched with 36 genes, including *pstC*, *pstA, BGLA_RS01225*, and *BGLA_RS26495*. Among these, *pstC* and *pstA* were associated with phosphate metabolism (Rao and Torriani, 1990). Other enriched pathways included polysaccharide metabolism (*glgX*, *kdsB*, *BGLA_RS30500*, etc.) and nitrogen compound transport (*BGLA_RS26495, BGLA_RS25180*, etc.).

KEGG pathway analysis revealed that the most significantly enriched pathways included two-component systems, quorum sensing, and bacterial secretion systems. The two-component system pathway showed the highest number of enriched genes, with 19 genes identified, including RNA polymerase sigma factor (*BGLA_RS06845/bgla_1g13750*), *cydB* (*BGLA_RS17470/bgla_1g35140*), cydX (BGLA_RS17475/bgla_1g35150), and heavy metal response regulator transcription factor (*BGLA_RS33570/bgla_2g2792*0). These findings suggest that the presence of the *bon* gene cluster is associated with differential expression of genes involved in secretion system. While this pattern may reflect potential adaptive differences, we are currently unable to infer a direct causal relationship.

Furthermore, *cydB* and *cydX* are primarily associated with electron transport under low-oxygen conditions, providing possible evidence that isolates containing the *bon* gene cluster mount specific responses to environmental stress for improved survival.

### 3.7 Metabolomic analysis of extracellular metabolic phenotypes in BA-producing and non-producing strains

Since BA was extracellular secondary metabolite, the metabolomic analysis was also conducted to further investigate the discriminations in extracellular metabolic phenotypes between BA-Producing strains (T group) and non-BA-producing strains (NT group). Before data analysis, the data quality and the robustness of the analytical method was evaluated. Over 80% (87.2% for T group and 83.4% for NT group) of the ion features possessed relative standard deviation (RSD) values≤35%, providing evidence for the robustness of analytical method. Meantime, unsupervised PCA model for the whole dataset was generated to explore the clustering trend of all the samples. As shown in Supplementary Figure S7A, not only the QCs (Quality Control samples), but also the T group and NT group were tightly clustered in PCA scores scatter plots, indicating good stability of analytical method. Significant discriminations in metabolic phenotypes between T and NT groups were observed by OPLS-DA models (R2Y = 0.997 and Q2 = 0.978, Supplementary Figure S7B). Furthermore, permutation tests of 200 cross-validation were performed to validate the OPLS-DA model, and the results suggested high goodness of fit and good predictive capability of the constructed models (Supplementary Figure S7C).

**Figure 6:**
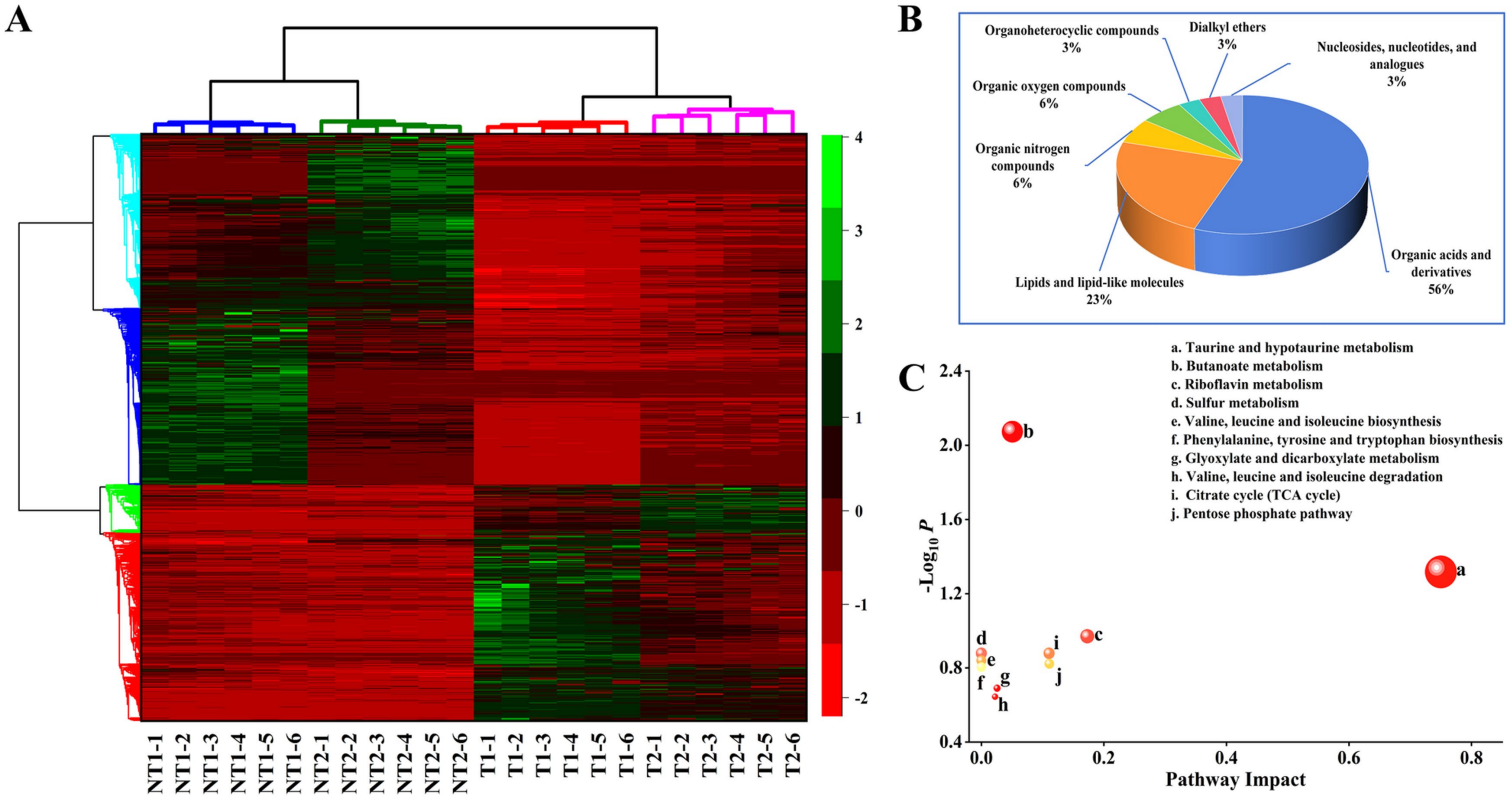
Differential metabolites analysis. **(A)** A heatmap showing the levels of differential metabolites across BA-producing and non-BA-producing groups, with two samples per group and six biological replicates per sample. The colors from green to red indicate higher levels of metabolites. **(B)** Pie charts showing the HMDB compound classification of differential metabolites. **(C)** Metabolic pathway enrichment and topological analysis of differentially expressed metabolites.

In total, 1,693 differential feature were selected after the filter of VIP value, FC value and adjusted-*p* value, which could be considered as the potential differential metabolites. As shown in Figure 6A, the samples of T and NT group were clustered accordingly based on the selected differential features, and significant difference in color distribution was observed across different groups. The volcano plots was subsequently depicted on the basis of adjusted-p and fold change (FC) values, where the red and green dots represented differential feature with adjusted-*p* < 0.05 and FC > 2 (or < 0.5) (Supplementary Figure S7D). After searching several databases for these features, 262 compounds were annotated. Among them, 34 compounds were identified by the MS/MS spectra. As illustrated in Figures 6B,C the identified metabolites mainly consisted of organic acids and derivatives and lipids and lipid-like molecules.

### 3.8 Multi-omics guided design of TaqMan probes

**Figure 7:**
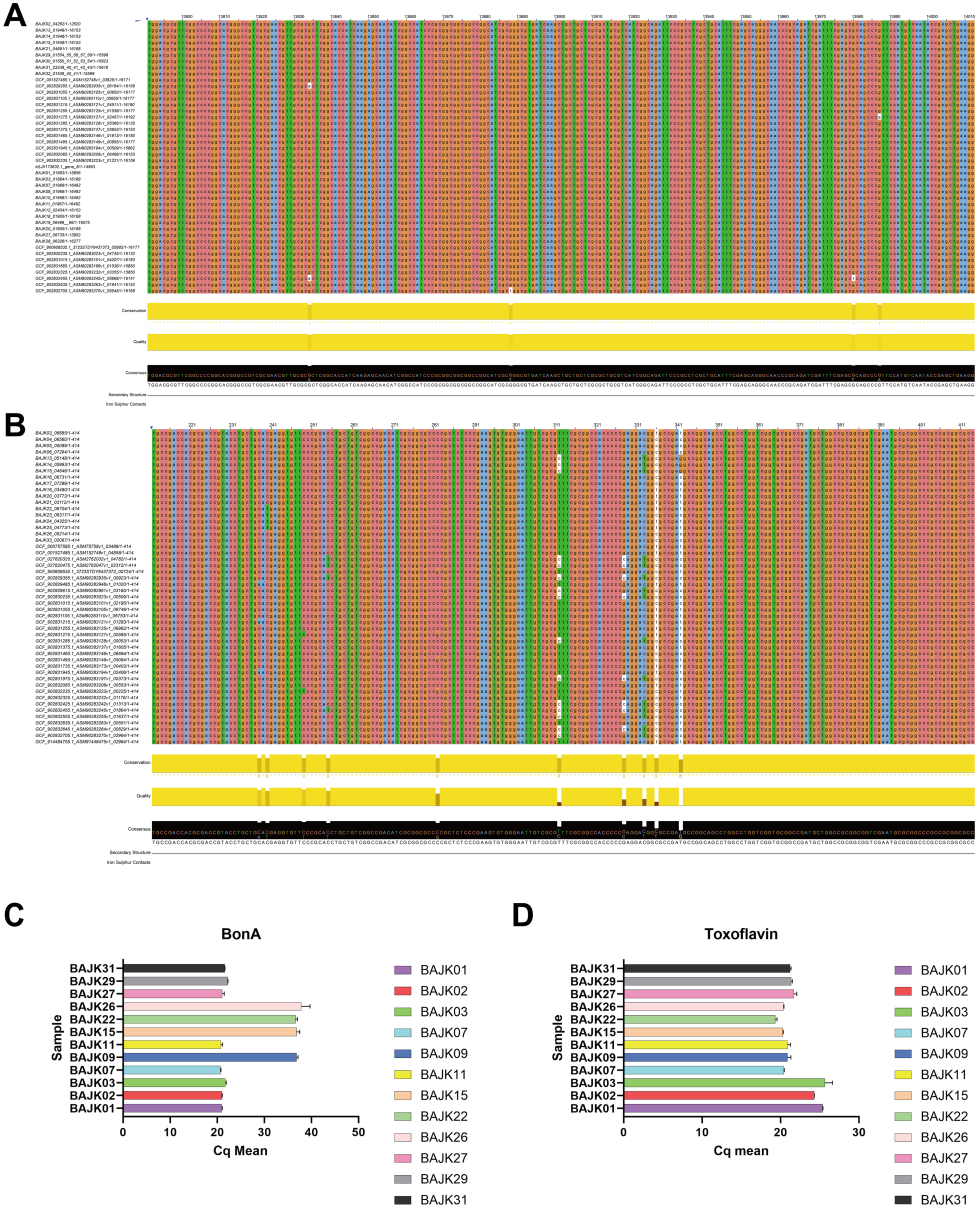
Multiple sequence alignments and qPCR results for detection genes. **(A)** Multiple sequence alignment of partial sequences of the *bonA* gene used for the detection of BA. This panel illustrates the alignment of sequences across different strains to identify conserved regions targeted by qPCR primers. **(B)** Multiple sequence alignment of partial sequences of the *tofI* gene used for the detection of Toxoflavin. This alignment is used to verify the conservation and suitability of target regions for primer design. **(C)** qPCR results for the *bonA* gene. A Cq (quantification cycle) value ≥ 36 indicates no gene expression, while a Cq value < 30 indicates gene expression. This graph displays the expression levels of *bonA* across tested samples, demonstrating the presence or absence of BA synthesis capability. **(D)** qPCR results for the *tofI* gene. A Cq value < 30 signifies gene expression. The graph presents the expression data for *tofI*, verifying the active synthesis of Toxoflavin in the tested strains.

To better prevent poisoning incidents, we designed TaqMan quantitative real-time PCR (qPCR) probes targeting the *bonA* gene within the bon gene cluster and the *tofI* gene that regulates toxoflavin synthesis, aiming to distinguish toxic-producing from non-toxic strains. These designs were based on comprehensive genomics and transcriptomics analyses. Previously, we identified *B. gladioli* strains harboring the *bon* gene cluster using blastp and constructed a phylogenetic tree (Figure 1). The presence or absence of the *bon* gene cluster clearly divided *B. gladioli* into two distinct clades. Further synteny analysis suggested that the *bon* gene cluster is highly conserved (Supplementary Figure S2), providing a reliable target region for primer design. Leveraging the conservation characteristics revealed by genomics, we identified stable regions of the *bonA* and *tofI* genes through multiple sequence alignment (Figures 7A,B), which ultimately enabled the design of specific TaqMan qPCR probes for differentiating toxic and non-toxic strains. Transcriptomics data further validated the efficacy of the target regions: analysis of BAM files from relevant strains showed high-abundance sequencing read coverage in the regions of the *bonA* and *tofI* genes targeted by our probes and primers (Supplementary Figures S8A,B). This indicates stable expression of these regions at the transcriptional level, ensuring the sensitivity and specificity of qPCR detection.

We measured the BA concentrations of 34 *B. gladioli* strains using UPLC-MS/MS. To validate the effectiveness of these primers, we randomly selected 12 strains from the 34 *B. gladioli* strains. The concentration detection results showed that 8 strains produced BA, while 4 strains did not. In the qPCR assay with DNA dilutions, 8 samples (BAJK31, BAJK29, BAJK27, BAJK11, BAJK07, BAJK03, BAJK02, BAJK01) exhibited Cq values of less than 30, indicating *bonA* gene expression; whereas BAJK26, BAJK22, BAJK15, and BAJK09 had Cq values greater than 36, confirming the absence of *bonA* gene expression (Figure 7C). These findings perfectly correlate with the presence or absence of BA compounds in the 12 *B. gladioli* samples, validating the design of the probes and primers. In contrast to the *bonA* gene, all 12 strains exhibited *tofI* gene expression (Figure 7D), suggesting that the production of BA and Toxoflavin in *B. gladioli* is neither mutually exclusive nor interdependent.

Additionally, we conducted sensitivity and specificity assays for the probe to assess its efficacy. The results are presented in Supplementary Figure S9. Supplementary Figure S9A suggests the probe’s exceptional sensitivity, achieving the threshold within 30 cycles despite dilution to a concentration of 10^−2^. The specificity experiment utilised three known toxin-producing strains, three non-toxin-producing strains, and one *Pseudomonas aeruginosa* strain. Results indicate that while amplification was completed within a low number of cycles for the three toxin-producing strains, the remaining four strains exhibited elevated Cq values (Supplementary Figure S9B).

## 4 Discussion

*Burkholderia gladioli* is a pathogen capable of infecting both plants and humans (Valvano et al., 2005; Yuan et al., 2024), occupying diverse ecological niches and exhibiting distinct biological characteristics. Even within the same species, different strains display variable phenotypes, largely attributable to genomic diversity (Lee et al., 2021). Comparative genomic studies have indicated that bacterial adaptation to different environments is primarily driven by gene gain/loss or genome expansion/contraction (Dutta and Paul, 2012). Specifically, strains of the same species may differ in their pathogenicity toward plants or humans; some produce metabolites and toxins that cause severe infections, whereas others are non-toxic or exhibit reduced virulence. For instance, *B. gladioli* synthesizes numerous secondary metabolites, including toxoflavin and BA (Anwar et al., 2017; Liu, 2009), though not all strains harbor the ability to produce these compounds (Lee et al., 2016; Moebius et al., 2012). Similarly, these strain-specific functions likely contribute to enhanced environmental adaptation.

BA has been confirmed as highly hazardous to humans, capable of inducing fatal respiratory toxicity, and multiple studies have linked this toxicity to the BA biosynthetic gene cluster (Han et al., 2023; Moebius et al., 2012). Acquisition of gene clusters can enhance bacterial adaptation, and the co-localization of genes within clusters ensures coordinated expression, thereby improving functional efficiency and stability (Wiedenbeck and Cohan, 2011). We therefore hypothesize that the presence of the *bon* gene cluster may confer unique survival advantages to *B. gladioli* in certain ecological niches.

In this study, we conducted whole-genome analyses on 305 *B. gladioli* strains and classified them into BA-producing and non-BA-producing groups based on the presence of the *bon* gene cluster, followed by phylogenetic analysis. Previous studies have shown that all *B. gladioli* strains can be grouped into three phylogenetic lineages, and BA-containing strains can be further subdivided into three sublineages (Jones et al., 2021; Peng et al., 2021). Our results confirm these findings and additionally reveal that some non-BA-producing and BA-producing strains belong to the same evolutionary branch. This distribution pattern may reflect one of the following scenarios: First, The common ancestor of *B. gladioli* acquired the *bon* gene cluster via HGT, which was then vertically inherited by its descendants, followed by the loss of this cluster via gene rearrangement in some strains during subsequent genetic processes. Second, the *bon* gene cluster was acquired independently via horizontal gene transfer (HGT) in specific strains. While the current data cannot fully distinguish between these two possibilities, the overall distribution pattern suggests that the gain and loss of the bon gene cluster exhibit a certain degree of phylogenetic correlation.

Virulence factors and antibiotic resistance genes are critical for the environmental adaptation of pathogenic bacteria and are commonly identified through whole-genome analysis. The VFDB database provides comprehensive information on bacterial virulence factors (Chen et al., 2005), whereas the CARD database catalogs antibiotic resistance genes and associated resistance mechanisms (Alcock et al., 2020). In our study, comparative genomics of BA-producing and non-producing strains revealed both conserved and variable features in virulence and resistance genes. For example, flagellar genes associated with motility were conserved across all strains, and the *Bpse_Omp38* resistance gene was universally present. Furthermore, we identified certain secretion system-associated virulence genes that may be linked to the presence of the *bon* gene cluster. We hypothesized that the acquisition of the *bon* gene cluster may have been accompanied by evolutionary changes in virulence factors—thereby enhancing the strains’ ability to adapt to diverse ecological niches—though experimental validation is required. However, no resistance genes potentially associated with the *bon* gene cluster were identified, This finding may suggest a lack of direct linkage between the bon gene cluster and antibiotic adaptation, though further research is needed to confirm this. We hypothesize that its evolutionary function may be more focused on bacterial ecological adaptation or host-pathogen interactions, rather than conferring a survival advantage under antibiotic pressure. This hypothesis is supported by the subsequent transcriptomic analysis results, which revealed an association between the *bon* gene cluster and secretion system-related genes. Nevertheless, further studies will be required to verify this.

Transcriptomic and metabolomic analyses are essential for investigating bacterial physiological functions under diverse conditions. By integrating transcriptomic and metabolomic data, we explored whether the *bon* gene cluster influences the physiology, virulence, and environmental adaptation of *B. gladioli*. Transcriptome analysis revealed that differentially expressed genes were predominantly enriched in the type III secretion system (T3SS) pathway. Compared to non-BA-producing strains, strains harboring the bon gene cluster exhibited significantly elevated expression levels of T3SS-related genes, a finding further validated by qRT-PCR experiments. This represents a previously unreported discovery, suggesting that the Bon gene cluster may possess direct or indirect relevance to T3SS. T3SS is a complex apparatus that spans the inner membrane, periplasm, outer membrane, extracellular space, and host cell membrane, allowing direct translocation of effectors into the host cytoplasm to exert broad virulence functions (Deng et al., 2017; Gerlach and Hensel, 2007). Previous studies have shown that environmental factors such as temperature, pH, and osmolarity can regulate T3SS gene expression, maintaining outer membrane stability and enhancing virulence (Flores-Kim and Darwin, 2012). Under certain conditions, *B. gladioli* utilizes T3SS to eliminate fungi and exploit them as a carbon source (Swain et al., 2017), reflecting how BA-producing strains may leverage T3SS to improve environmental stress adaptation and survival in complex habitats.

Previous studies have suggested that organic acids and lipids promote BA biosynthesis (Anwar et al., 2017; Garcia et al., 1999; Rohm et al., 2010), and these metabolites play essential roles in bacterial physiology as precursors for secondary metabolite synthesis (Yan et al., 2024), such as streptomycin. Moreover, secretion of most secondary metabolites is mediated by transport proteins (Martín et al., 2005), which interact with membrane lipids to facilitate export (Nikaido, 1968; Stieger et al., 2021). Our untargeted metabolomic analysis revealed that BA-producing strains synthesized higher levels of organic acids and lipids compared to non-BA-producing strains, supporting the conclusion that BA biosynthesis and secretion require substantial metabolic input.

Similar to BA, toxoflavin is another highly toxic compound affecting both mammals and plants (Choi et al., 2023; Lee et al., 2016). To mitigate the risks posed by these toxins, we developed rapid detection probes targeting the primary biosynthetic genes of BA and regulatory gene of toxoflavin (*bonA* and *tofI*). This cost-effective method is suitable for food safety monitoring and substantially reduces detection expenses.

In summary, our study applied multi-omics approaches to investigate differences in environmental adaptation between BA-producing and non-BA-producing *B. gladioli* strains and developed a convenient method for distinguishing toxigenic strains. The contribution of BA to *B. gladioli* environmental adaptation is complex and requires further investigation to elucidate the underlying regulatory mechanisms. Future studies will focus on functional genomics and transcriptional regulation, establishing bon knockout strains to further elucidate the molecular mechanisms by which key genes mediate strain responses to environmental stress.

## 5 Conclusion

This study analyzed the genetic diversity of 305 *B. gladioli* strains isolated up to September 2023, revealing that the *bon* gene sequence is relatively conserved. Regarding the origin of its acquisition, multiple evolutionary events are plausible, such as gene loss caused by genetic rearrangement or horizontal gene transfer (HGT). Virulence factor analysis indicated that both BA-producing and non-BA-producing strains exhibit broadly similar virulence profiles, although certain virulence genes associated with pyruvate metabolism, nitrate reduction, and polyketide synthase were more commonly present in BA-producing strains. Furthermore, antibiotic resistance gene prediction suggests that *Burkholderia gladioli* harbors resistance genes against quinolones and *β*-lactam antibiotics. Additionally, we conducted multi-omics analysis using four *B. gladioli* strains (two BA-producing and two non-BA-producing) to explore the impact of BA on the environmental adaptability of *B. gladioli* through transcriptomics and metabolomics. The results showed that BA-producing strains exhibited significantly higher expression of genes related to the type III secretion system compared to non-BA-producing strains, and BA-producing strains also produced significantly higher levels of organic acids, their derivatives, and lipid molecules. Finally, we developed a rapid detection method to distinguish toxin-producing strains by detecting the bonA and tofI genes, providing a simple way to identify whether *B. gladioli* strains are toxic to humans. Our study offers valuable insights into public health safety and lays the foundation for further research on the role of BA in enhancing the environmental adaptability of *B. gladioli.*

## Data Availability

The datasets presented in this study can be found in online repositories. The names of the repository/repositories and accession number(s) can be found at: https://www.ncbi.nlm.nih.gov/genbank/, PRJNA1206991; https://www.ncbi.nlm.nih.gov/, PRJNA1191178.

## Footnotes

1
https://github.com/wdecoster/NanoFilt

2
https://github.com/moshi4/pyGenomeViz

3
https://github.com/ncbi/amr
