# Correction: Integrative omics analysis reveals distinct adaptations of bongkrekic acid producing *Burkholderia gladioli pathovar cocovenenans* strains

**DOI:** 10.3389/fmicb.2026.1816526

**Published:** 2026-03-12

**Authors:** Jiale He, Lingguo Zhao, Yunke Sun, Qingliang Chen, Jiashu Li, Jun Chen, Yingdan Zhang, Liang Yang, Yang Liu, Lei Lei

**Affiliations:** 1 Medical Research Center, Southern University of Science and Technology Hospital, Shenzhen, China; 2 Joint Laboratory of Guangdong-Hong Kong Universities for Vascular Homeostasis and Diseases, Department of Pharmacology, School of Medicine, Southern University of Science and Technology, Shenzhen, China; 3 Bao'an District Center for Disease Control and Prevention of Shenzhen City, Shenzhen, China; 4 Shenzhen Third People's Hospital, The Second Affiliated Hospital of Southern University of Science and Technology, Shenzhen, China; 5 National Clinical Research Center for Infectious Disease, Shenzhen Third People's Hospital, Shenzhen, China

**Keywords:** bongkrekic acid, comparative genomic analysis, environmental adaptability, fluorescent probe detection method, omics analysis

In the published article, there was a mistake in the author list. Author Jiashu Li was omitted as an author in the published article.

The correct author list reads:

Jiale He^1,2‡^, Lingguo Zhao^3‡^, Yunke Sun^2,4,5†^, Qingliang Chen^3^, Jiashu Li^2^, Jun Chen^4^, Yingdan Zhang^4,5†^ Liang Yang^2,4*^, Yang Liu^1*^, Lei Lei^3*^

^1^Medical Research Center, Southern University of Science and Technology Hospital, Shenzhen, China

^2^Joint Laboratory of Guangdong-Hong Kong Universities for Vascular Homeostasis and Diseases, Department of Pharmacology, School of Medicine, Southern University of Science and Technology, Shenzhen, China

^3^Bao'an District Center for Disease Control and Prevention of Shenzhen City, Shenzhen, China

^4^Shenzhen Third People's Hospital, The Second Affiliated Hospital of Southern University of Science and Technology, Shenzhen, China

^5^National Clinical Research Center for Infectious Disease, Shenzhen Third People's Hospital, Shenzhen, China

The Author Contributions Statement has been corrected to read:

Jiashu Li: Writing – review & editing.

In the published article, an incorrect **Funding** statement was provided. The funding statement was displayed as “Natural Science Foundation of Shenzhen”. The correct funder is “Shenzhen Science and Technology Program”.

In the published article, there was a mistake in the **Supplemental material**. The supplemental material Supplementary_fig.docx was omitted. The correct file have now been published.

A correction has been made to the section [*3.7 Metabolomic analysis of extracellular metabolic phenotypes in BA-producing and non-producing strains*]:

“As shown in Figure 6A, the samples of T and NT group were clustered accordingly based on the selected differential features, and significant difference in color distribution was observed across different groups. The volcano plots was subsequently depicted on the basis of adjusted-*p* and fold change (FC) values, where the red and green dots represented differential feature with adjusted-*p* < 0.05 and FC > 2 (or < 0.5) (Supplementary Figure S7D).”

The original version of this article has been updated.

