# Supplementary material for: Integrative omics analysis reveals distinct adaptations of bongkrekic acid producing Burkholderia gladioli pathovar cocovenenans strains
Source: Front Microbiol. 2026 Feb 5;16:1712709. doi: 10.3389/fmicb.2025.1712709 (PMC12916611; doi:10.3389/fmicb.2025.1712709)
Supplement: Supplementary file 1 [file Table_1.DOCX]

**Integrative omics analysis reveals distinct adaptation of Bongkrekic Acid producing *Burkholderia gladioli pathovar cocovenenans* strains**

Jiale He^1, 2‡^, Lingguo Zhao^3‡^, Yunke Sun^2,4,5†^, Qingliang Chen^3^, Jiashu Li^2^, Jun Chen^4^, Yingdan Zhang^4,5†^ , Liang Yang^2, 4*^, Yang Liu^1*^, Lei Lei^3*^


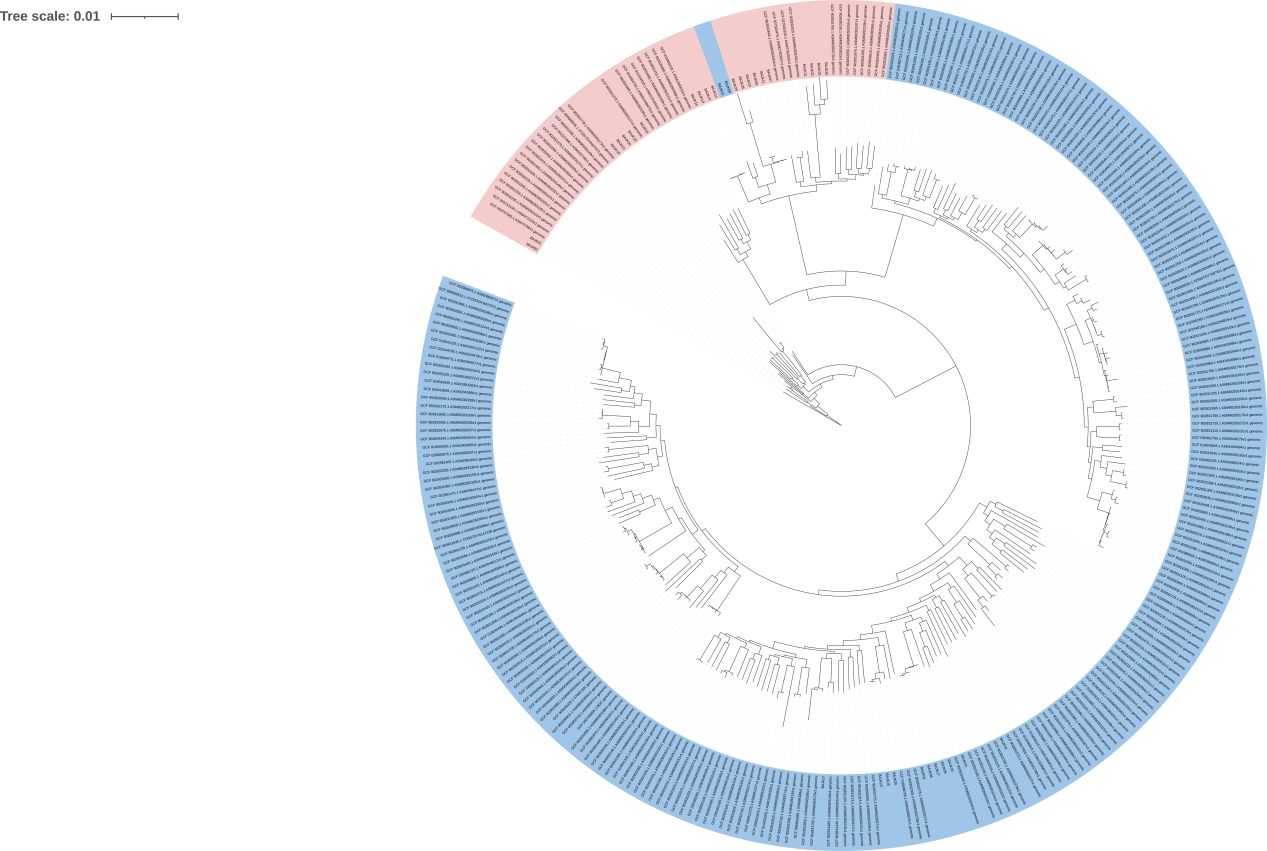
**Figure S1. Core-genome phylogeny excluding the bon gene cluster.** Maximum-likelihood phylogeny reconstructed from concatenated core-gene alignments after removal of the bon gene cluster and other annotated mobile genetic elements.Strains carrying the *bon* cluster are shown in pink, and strains lacking the cluster are shown in blue.


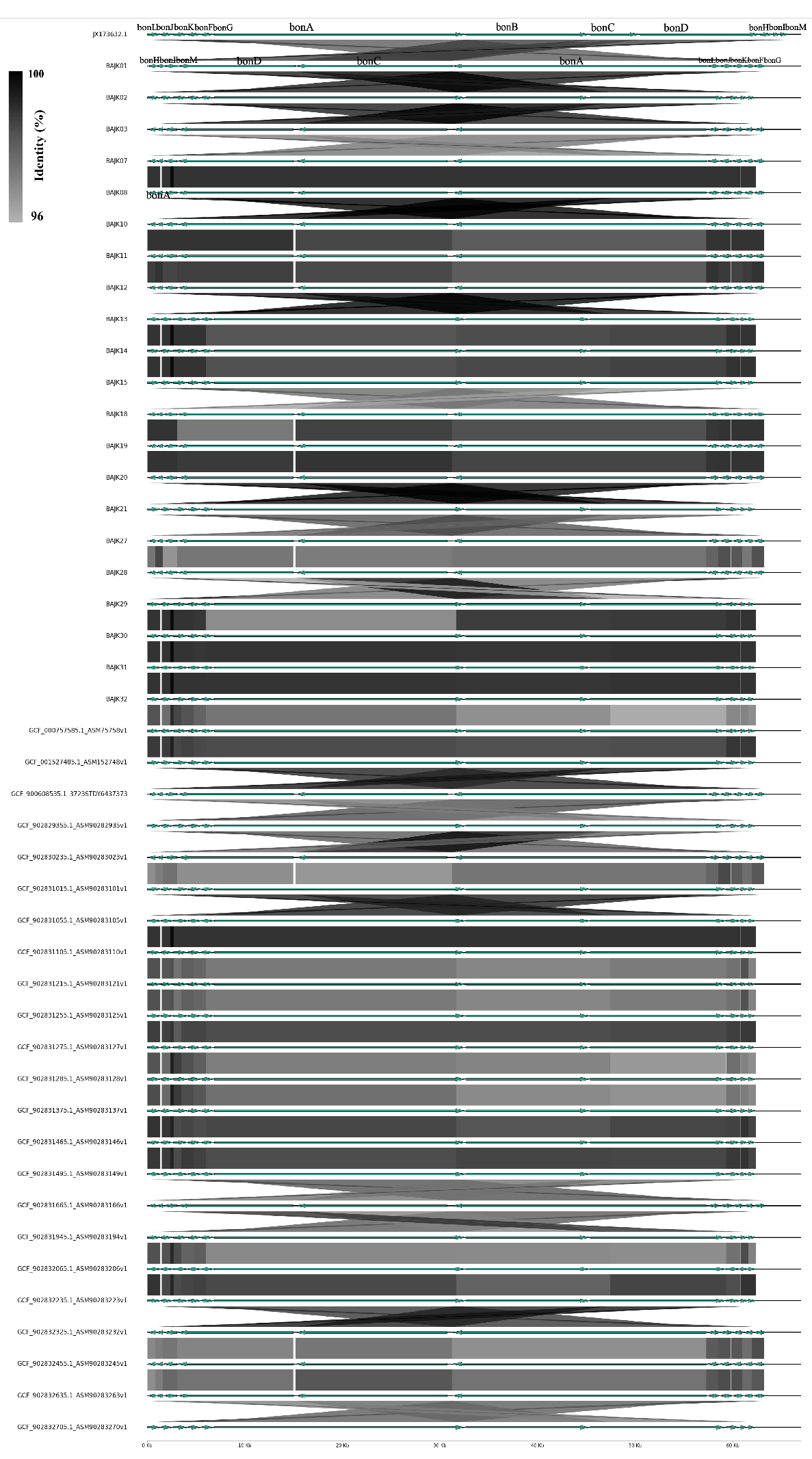


**Figure. S2** A total of 305 *B. gladioli* strains were screened for the presence of the *bon* gene cluster, and the sequences of the *bon* gene cluster from these strains were extracted for Collinearity analysis.


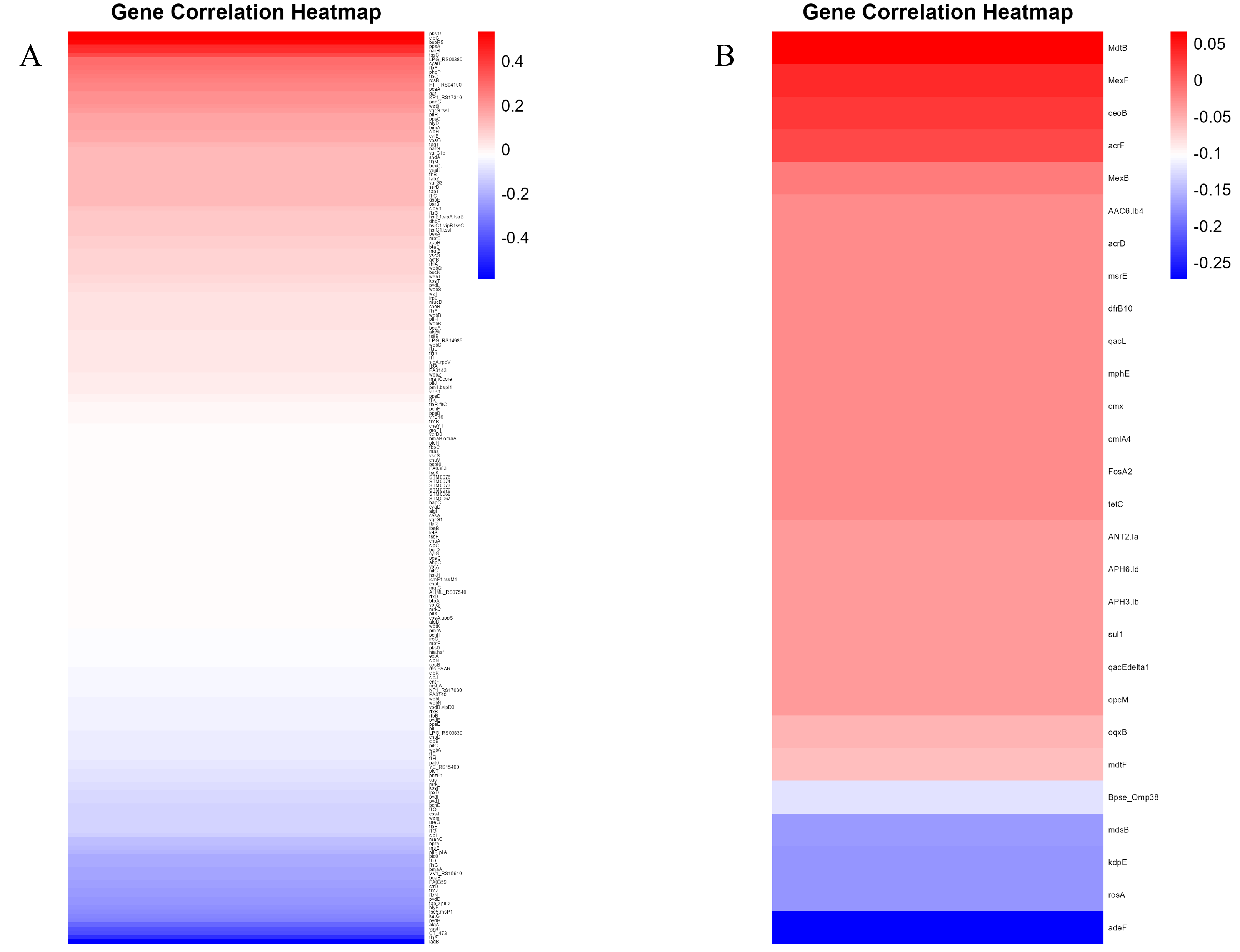


**Figure. S3.** Spearman correlation coefficient was used to assess the relationship between *bon* gene cluster and genes, presented as a heatmap. **A** shows the correlation between *bon* gene cluster and virulence factors, while **B** illustrates the correlation between *bon* gene cluster and resistance genes. Positive correlations are represented in red, and negative correlations in blue.


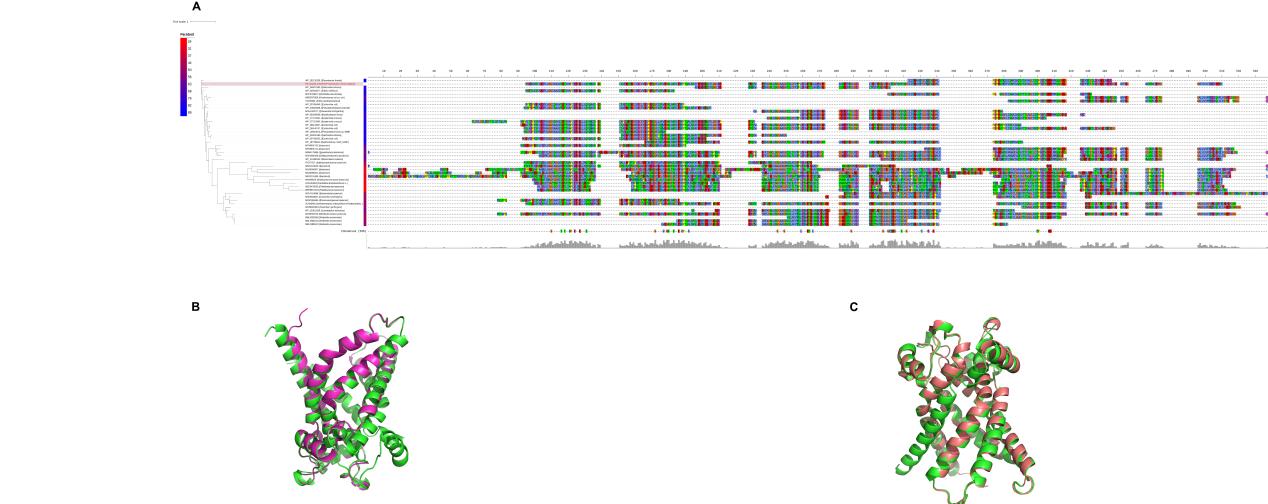


**Figure. S4 A.** Phylogenetic tree of SLC25A4 homologs, with human SLC25A4 highlighted in pink. The heatmap indicates the level of homology, with blue and red representing low and high homology, respectively. On the right, the multiple sequence alignment of 50 homologous proteins is displayed.  **B.** 3D structure alignment of WP_266143627.1 with SLC25A4 protein. **C.** 3D structure alignment of WP_266145757.1 with SLC25A4 protein.


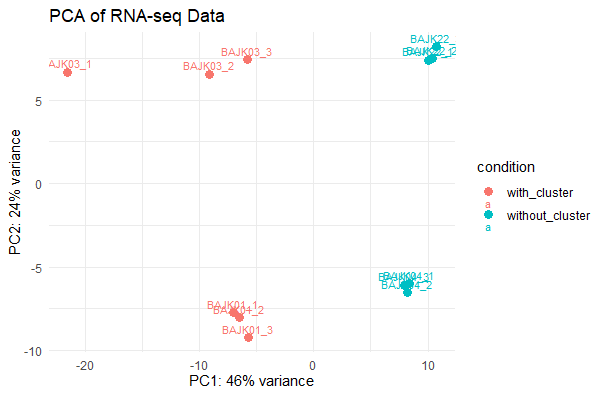


**Figure. S5** PCA plot of RNA-seq data, showing the presence or absence of the *bon* gene cluster. Pink represents strains with the *bon* gene cluster, while blue indicates strains without the *bon* gene cluster.


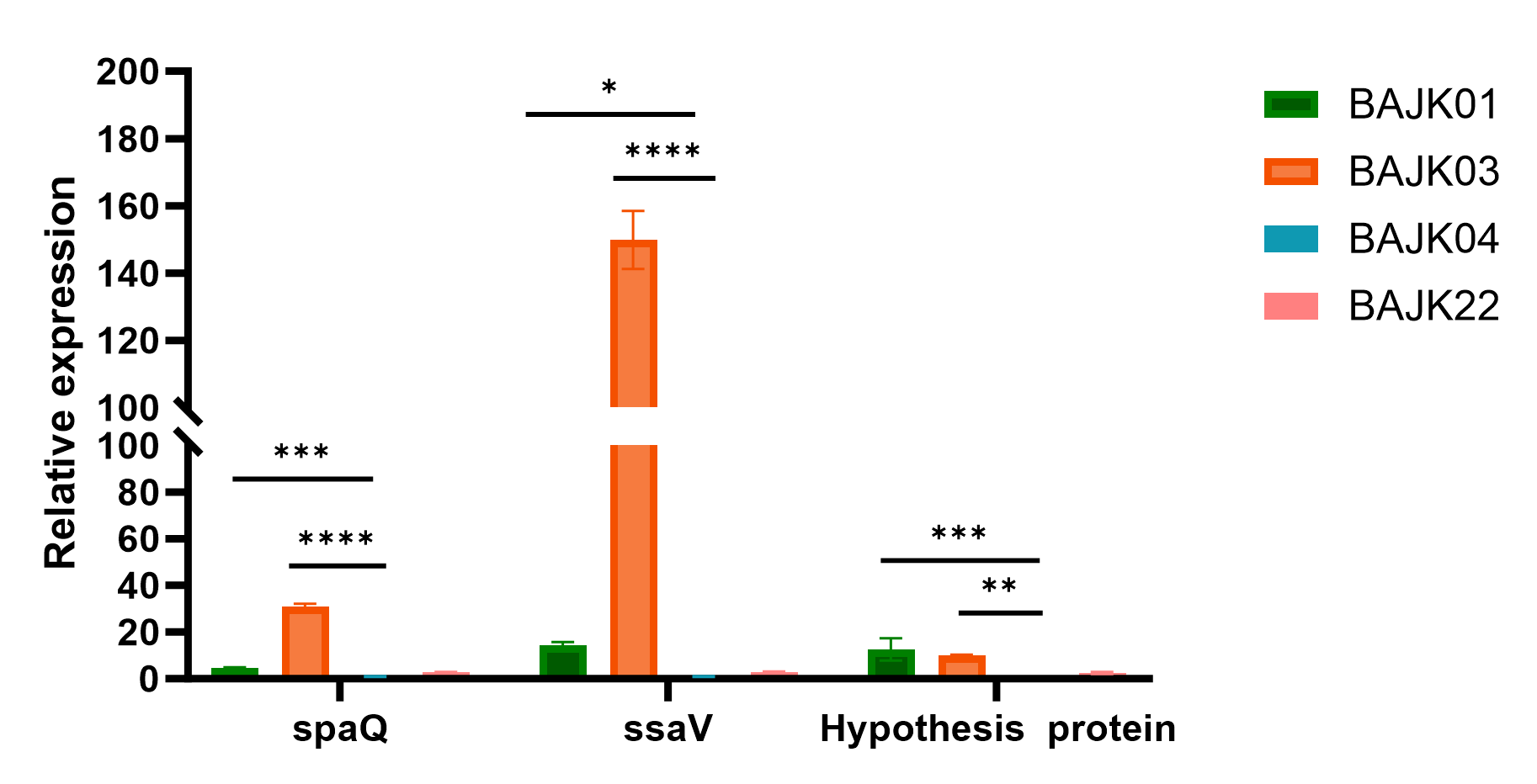


**Figure S6.** RT-PCR validation of differentially expressed genes related to the type III secretion system in BAJK01, BAJK03, BAJK04, and BAJK22. *P < 0.05, ***P < 0.001, ****P < 0.0001.

**
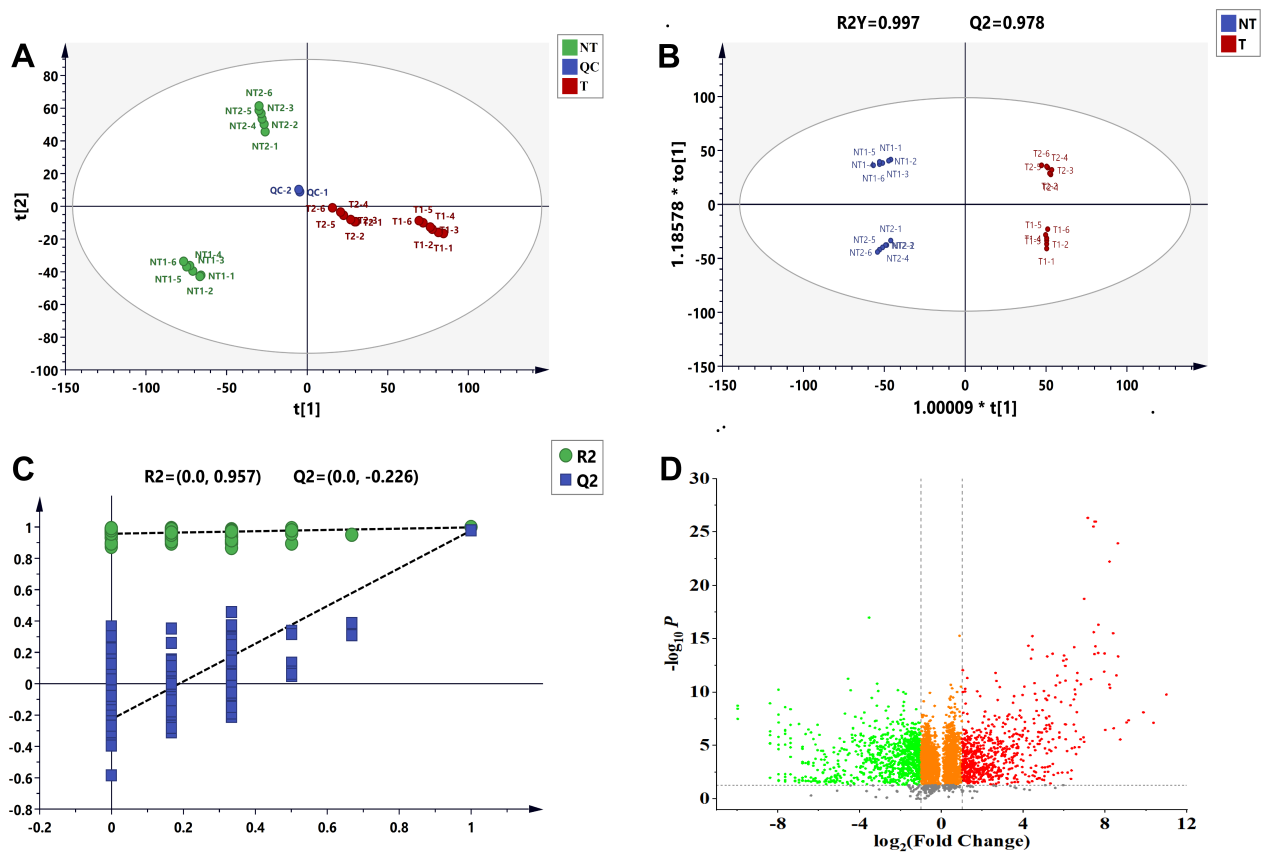
**

**Figure S7.** UHPLC-Q-Orbitrap HRMS-based untargeted metabolomic analysis. **A.** Principal Component Analysis (PCA) score plot.Different colors represent different sample types (NT, T, and QC). Ellipses denote 95% Hotelling’s T² confidence intervals, used to assess sample distribution and data stability. Orthogonal Partial Least Squares Discriminant Analysis (OPLS-DA) score plot. **B.** Model fit (R²Y) and predictive power (Q²) are annotated in the figure.**C.** Permutation test for the OPLS-DA model. Green and blue points represent R² and Q² values, respectively, with dashed lines indicating their trend. **D.** Volcano plot of differentially expressed metabolites. The x-axis represents log₂(Fold change) of metabolites, while the y-axis shows −log₁₀(P-value). Different colors denote metabolites that are up-regulated, down-regulated, or show no significant difference between the two groups.


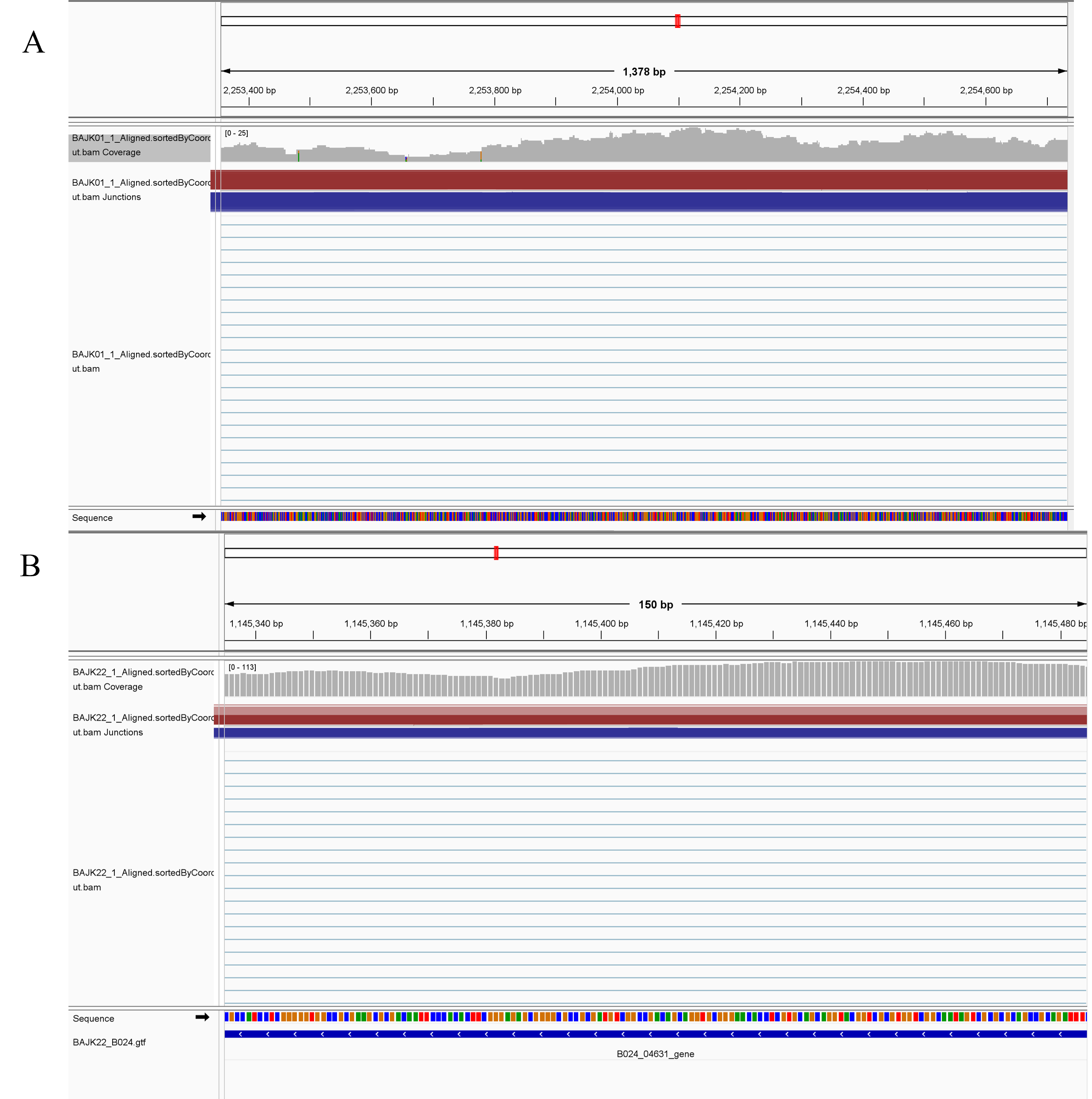


**Figure S8.** The sequencing read coverage of the target sequences for probes and primers in transcriptome sequencing. **A.** The sequencing read coverage of the *bonA*sequence targeted by the probe and primer (gray). **B.** The sequencing read coverage of the *tofI*sequence targeted by the probe and primer (gray).


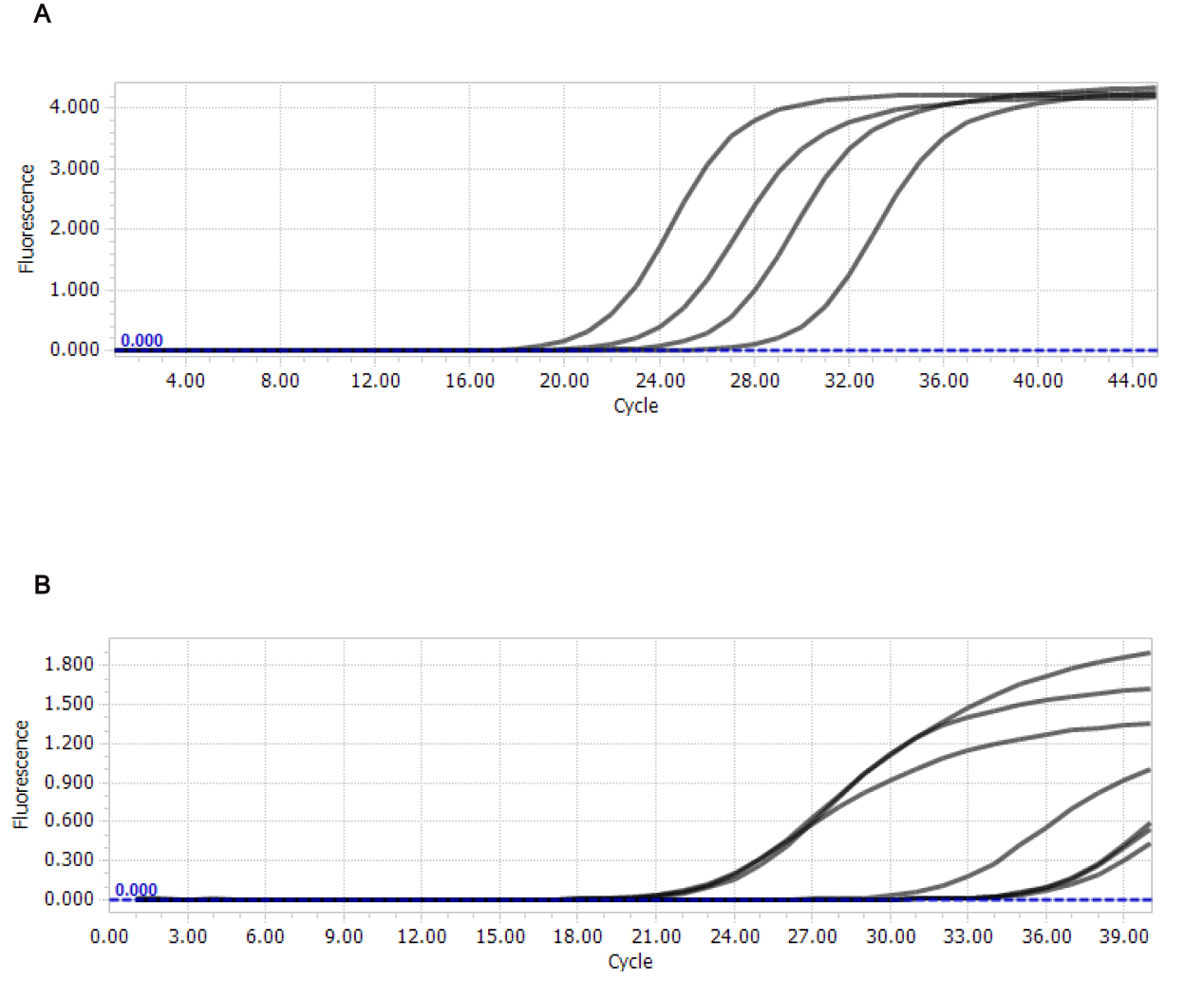


**Figure S9.** Sensitivity(**A**) and Specificity(**B**) Testing of *bonA* Probe Primers.
